# Malay Lexicon Project 3: The impact of orthographic–semantic consistency on lexical decision latencies

**DOI:** 10.1177/17470218241234668

**Published:** 2024-03-21

**Authors:** Mirrah Maziyah Mohamed, Debra Jared

**Affiliations:** Department of Psychology, University of Western Ontario, London, ON, Canada

**Keywords:** Psycholinguistics, morphology, visual word recognition, lexical decision, distributional semantics, orthographic–semantic consistency, Malay

## Abstract

Theories of word processing propose that readers are sensitive to statistical co-occurrences between spelling and meaning. Orthographic–semantic consistency (OSC) measures provide a continuous estimate of the statistical regularities between spelling and meaning. Here we examined Malay, an Austronesian language that is agglutinative. In Malay, stems are often repeated in other words that share a related meaning (e.g., *sunyi*/*quiet; ke-sunyi-an*/*silence; makan*/*eat; makan*-*an*/*foods*). The first goal was to expand an existing large Malay database by computing OSC estimates for 2,287 monomorphemic words. The second goal was to explore the impact of root family size and OSC on lexical decision latencies for monomorphemic words. Decision latencies were collected for 1,280 Malay words of various morphological structures. Of these, data from 1,000 monomorphemic words were analysed in a series of generalised additive mixed models (GAMMs). Root family size and OSC were significant predictors of decision latencies, particularly for lower frequency words. We found a facilitative effect of root family size and OSC. Furthermore, we observed an interaction between root family size and OSC in that an effect of OSC was only apparent in words with larger root families. This interaction has not yet been explored in English but has the potential to be a new benchmark effect to test distributional models of word processing.

## Introduction

Research on statistical regularities in visual word recognition has devoted considerable attention to the impact of spelling–sound correspondences on reading (e.g., Glushko, 1979; Jared, 2002; Jared et al., 1990; Siegelman et al., 2020; Treiman et al., 1995; Waters & Seidenberg, 1985; Yap & Balota, 2009). This research has shown that reading times for a word are faster if orthographic neighbours all have a similar pronunciation (e.g., *must* has similarly pronounced neighbours such as *dust*, *just*, and *rust*) than if some orthographic neighbours have a different pronunciation (e.g., *cheat* has two neighbours with a conflicting pronunciation—*great* and *sweat*). Words can also vary in how semantically similar they are to their neighbours. Spelling–meaning regularities have been studied to a lesser extent and primarily in the context of morphological processing in word recognition. Some English words (e.g., *bright*), share meaning with their neighbours (e.g., *brighter, brightest, brightly, brightness*), whereas other words (e.g., *whisk*) have some neighbours that do not share its meaning (e.g., *whisker, whiskey*). Marelli et al. (2015) developed a measure of orthographic–semantic consistency (OSC) that provides a continuous estimate of the extent to which a word’s meaning is related to its orthographic neighbours. Such a measure can help researchers determine whether skilled readers pick up on spelling–meaning regularities from their experience with print. Marelli et al. (2015), Siegelman, Rueckl, et al. (2022), and Hendrix and Sun (2021) have shown that OSC predicted English lexical decision latencies. A broad goal of the present study is to extend their research to another language, Malay. To do so, we expanded on an existing large Malay word database by computing OSC estimates for a large set of words and explored the impact of OSC on lexical decision latencies in Malay readers.

Before describing our present study, we first discuss the relevant theoretical accounts and the current evidence on the impact of orthographic-semantic regularities on decision latencies, and then provide a brief introduction of the Malay language.

### Theoretical accounts of spelling–meaning regularities

Of most theoretical relevance to the present study is the distributed approach to morphological processing (Baayen et al., 2019; Marelli & Baroni, 2015; Plaut & Gonnerman, 2000; Rueckl & Raveh, 1999). These theories propose that readers are sensitive to statistical co-occurrences between spelling and meaning. In these views, there is no explicit representation of morphemes. The representations of a word’s form and meaning are shaped by distributional properties such as the statistical regularities between spelling and meaning.

Distributed accounts of morphological processing are often implemented computationally in the form of connectionist models (for a review see Stevens & Plaut, 2022). In connectionist models, representations of words are encoded over sets of orthographic, phonological, and semantic units. Each set of units has weighted connections to the other two sets that adjust over time with learning. Simulations have shown that the models pick up on the structure of the words on which they are trained, such as the statistical relationships between spelling and meaning. For example, Rueckl and Raveh (1999) trained a three-layer neural network (with orthographic, hidden, and semantic layers) to map orthographic representations onto semantic representations. Two different artificial languages were used as the training vocabulary. One language contained morphological regularity (i.e., letter patterns were a reliable cue to meaning) and the other language did not contain morphological regularity. The authors found that considerably less training was required for the model to learn form-to-meaning mappings when the model was trained on the language that contained morphological regularity than when the model was trained on the language that did not contain morphological regularity. When they examined the hidden layer representations in the models, there was greater similarity between representations for a root word and its morphological relatives in the former model than between word pairs of comparable orthographic similarity (but no shared meaning) in the latter model. That is, there was a clustering of morphological relatives in the hidden layer of the model that was trained on a language with morphological regularity even though no explicit morphological information was given to the model. Furthermore, in that model, stems and suffixes were represented by distinct hidden layer subpatterns, indicating that multimorphemic words had a componential structure. These findings provide evidence for the assumption of the connectionist view that morphological structure is an emergent property of the structure in the mapping between orthography and meaning.

Similarly, the discriminative lexicon model (DLM; e.g., Baayen et al., 2019) consists of weighted connections between spelling and meaning. In the DLM, the spelling and meaning of a word are each represented by a set of high-dimensional vectors. The model creates a mapping matrix between these two types of vectors using a linear discriminative learning algorithm. In discriminative learning, whenever associations between an orthographic pattern and a semantic pattern are strengthened, the association between that orthographic pattern and other meanings are weakened at the same time. The mapping matrix is then used to predict a semantic vector, given an orthographic vector for a word. Accuracy of the model is assessed by computing a correlation between a predicted semantic vector and the target (i.e., correct) vector. Correlations between a predicted semantic vector and a target vector are affected by the consistency of the mapping from the orthographic form to semantics. If the model has to learn to associate components of the orthographic representation with a wide range of semantic features (as in *whisk*, *whiskey*, and *whisker*), the strength of the relevant connection weights for each word will be lower than for an orthographic pattern that is mapped to a narrower range of semantic features. As a consequence, the correlation between the predicted and target semantic vectors is expected to be smaller in the former case than in the latter.

In all these models, despite varying in some model-specific details, readers are assumed to pick up on statistical regularities between spelling and meaning to some degree. In the following section, we discuss behavioural evidence from the existing literature on the impact of spelling–meaning consistency on lexical decision latencies.

### Behavioural evidence of spelling–meaning regularities

#### Root family size

Morphological root family size, a notable predictor known to influence word recognition, encompasses statistical regularities between spelling and meaning (e.g., *teach, teacher, teaching*). Root family size is defined in terms of the number of words that share the same root. For example, the root family size of the Malay root word *ajak*/*invite* is three because *ajak* appears three times in the Malay Lexicon Project (MLP) database (i.e., *ajak*/*invite, ajakan/*invitation, *mengajak*/*to invite*). It is well-established that root family size exerts a facilitative effect on lexical decision latencies across a variety of languages such as in English (Ford et al., 2010; Sánchez-Gutiérrez et al., 2018), Danish (Balling & Baayen, 2008), Dutch (Bertram et al., 2000; De Jong et al., 2000; Dijkstra et al., 2005), Estonian (Lõo et al., 2018), Finnish (Moscoso del Prado Martín et al., 2004), and Hebrew (Moscoso del Prado Martin et al., 2005). Words that belong to a larger root family size are responded to faster than those that have smaller families. This facilitative effect of root family size was also observed in morphologically complex words in Malay, particularly in lower frequency words (Maziyah Mohamed et al., 2023; Maziyah Mohamed & Jared, 2024). The composition of family members in Malay is primarily derived word relatives, whereas in other languages family members include many compound words.

#### Root family frequency

Root family frequency is a token-count measure of root family size. The effect of family frequency is much more nuanced than that of family size. A facilitative effect of root family frequency on lexical decision latencies has been found in several languages under certain circumstances. Baayen et al. (2007) found an effect of family frequency in English for lower frequency words. In French, Colé et al. (1989) found an effect of family frequency in suffixed words but not in prefixed words, whereas Mailhot et al. (2020) did not find an effect of family frequency for suffixed words. Järvikivi et al. (2006) observed an effect of family frequency in Finnish only for suffixed words that did not have allomorphs. Schreuder and Baayen (1997) did not find an effect of family frequency when family size was accounted for, whereas other studies (Ford et al., 2010; Sánchez-Gutiérrez et al., 2018; Vannest et al., 2002) found an effect of family frequency above and beyond family size. In our previous studies of Malay (Maziyah Mohamed et al., 2023; Maziyah Mohamed & Jared, 2024), we found a significant effect of family frequency, but when we tried to examine whether there was an effect of family frequency with family size in the model, the model concurvity statistics were unacceptably high. Family frequency was highly correlated with whole-word frequency which was also in our models.

#### OSC

As previously noted, OSC refers to the extent to which a stem’s meaning is related to its orthographic neighbours. A fundamental difference that we would like to emphasise to readers in the measures of OSC and root family size is in the types of neighbours that are captured by each measure. In the calculations of OSC, the neighbours of a target word (e.g., *ajar*—teach) contain morphological neighbours that share meaning (e.g., *aja* ***r*** *an*—teachings) as well as neighbours that are purely orthographic and do not map onto similar meanings (e.g., *fajar*—dawn). The calculations of root family size (i.e., count of words that share the same root), however, include only morphological neighbours. Therefore, the critical difference between the two measures is that orthographic neighbours of a target word that do not share meaning are included in the calculations of OSC, but not in the calculations of root family size.

In a preliminary study, Marelli et al. (2015) treated orthographic-semantic consistency as a binary variable. They had noted that in masked morphological priming studies, target words that were in the truly suffixed (transparent) prime condition (e.g., *soften-SOFT* vs. *heroic-SOFT*) had shorter lexical decision latencies than target words in the pseudo-suffixed (opaque) prime condition (e.g., *planet-PLAN* vs. *editor-PLAN*) on both the related and unrelated trials. If one considers the related prime as one of the target’s neighbours, then targets used in the opaque condition had at least one inconsistent neighbour (e.g., *planet* is unrelated in meaning to *plan*), whereas the targets used in the transparent condition had at least one consistent neighbour. Marelli et al. examined whether responses to these two sets of target words differed when they were not in a priming experiment. They extracted lexical decision latencies from the British Lexicon Project (Keuleers et al., 2012) for 325 words that had been used as target words in a number of published masked morphological priming studies. The authors observed a facilitative effect of transparency. That is, words that had been targets in the transparent condition produced shorter response times than words that had been targets in the opaque condition. Marelli et al. proposed that the underlying effect of transparency is a result of the two sets of words varying in their orthographic reliability as a cue for a word’s meaning.

Marelli et al. (2015) then developed a continuous measure (i.e., OSC) that assesses the consistency of the mappings between spelling and meaning for a word with its neighbours. Neighbours were defined as other words that started with the same letter string (e.g., SEAT: *seats*, *seating*, *seated*, *seatless*, etc.). The meaning of a word was represented by a vector created using methods from distributional semantics (specifically, Latent Semantic Analysis (LSA); Landauer & Dumais, 1997). The approach assumes that the meaning of a word can be approximated by considering the way that a word co-occurs with other words in the lexicon (i.e., words that co-occur in similar contexts tend to share related meanings). Semantic vectors are derived from these co-occurrences. The semantic similarity between a word and one of its neighbours is calculated using the cosine of the angle between their two vectors. OSC was a frequency-weighted average semantic similarity between a word and each of its neighbours. We provide more details on the calculation of OSC in the “Method” section. Marelli et al. investigated their OSC measure using two datasets: one was the dataset used in their previous study, and the second was a much larger dataset of lexical decision latencies for over 1,800 words, also from the British Lexicon Project. In both sets of analyses, they found that OSC exerted a facilitative effect on decision latencies after frequency, length, and root family size were accounted for. The model that included OSC produced a better fit to the data from the first dataset than the model that included “transparency” as a categorical predictor. This finding of an effect of OSC even when root family size was included in the model provides some evidence that O-S mappings for orthographic neighbours other than morphological relatives influence decision latencies.

In a subsequent article, Marelli and Amenta (2018) revised their neighbourhood definition, relaxing the positional constraint to include all words that contained the word of interest instead of just those starting with that word (e.g., SEAT: *seats*, *seating*, *seated*, *seatless*, *reseat*, *unseat*, *nauseate*, etc.). Furthermore, they generated their semantic vectors using a prediction-based approach (or word embeddings model). The authors demonstrated that OSC calculated using this new procedure accounted for more variance in lexical decision latencies from a dataset of over 3,000 words extracted from the British Lexicon Project than their original OSC measure. Again, frequency, length, and root family size were included in the model. Subsequent analyses demonstrated that both the continuous bag of words (CBOW) and skip-gram prediction approaches to generating semantic vectors using the word2vec tool produced similar OSC values. In CBOW, the vector dimensions capture the extent to which a word is predicted by the contexts in which it appears, whereas skip-gram dimensions indicate how well contexts are predicted by the target word. Marelli and Amenta (2018) also explored a variety of ways of defining neighbours, including the closest *n* neighbours based on Levenshtein distance (*n* = 10, 20, or 30), and neighbours with a Levenshtein distance of *n* (*n* = 1, 2, or 3). None of these six neighbourhood definitions produced OSC values that were as predictive of lexical decision times as their definition of neighbours as words that embed the target word.

Siegelman, Rueckl, et al. (2022) raised a concern about the way OSC was calculated in Marelli and colleagues’ work. They pointed out that because the word itself was included in its neighbourhood, and the calculation of mean semantic similarity was frequency-weighted, their OSC measure is affected by the ratio between the frequency of the word and the frequency of its neighbours. This frequency–ratio captures more broadly various types of statistical structure across the O-P, O-S, and P-S mappings, as well as within each O, P, and S representations. Consequently, Siegelman et al. claimed that Marelli et al.’s OSC measure does not exclusively capture orthographic-semantic regularities between a target word and its neighbours. They developed an OSC formula that differs from Marelli’s in that their OSC estimates were type-based rather than frequency-weighted, that is, each word was counted once regardless of its frequency. Type-based OSC in their study was defined as the average semantic similarity between a word and each of its orthographic neighbours. Siegelman et al. defined orthographic neighbours as words with a Levenshtein distance of 1 from a word, that is, that differed by one letter through addition, subtraction, or substitution (e.g., SEAT: *seats*, *sat*, *set*, *eat*, *sea*, *feat*, *beat*, *heat*, *sent*, *seal*, *seam*, etc.) to maximise the number of words that had at least one neighbour. In their study, monosyllabic words and their corresponding behavioural data were extracted from the English Lexicon Project (ELP; Balota et al., 2007). The authors found a significant impact of type-based OSC on lexical decision latencies and accuracy, but not for word naming. Words that had a higher OSC were responded to faster and more accurately than words that had a lower OSC. Models included as control variables’ frequency, length, orthographic, and phonological neighbourhood sizes, but not root family size. In contrast to type-based OSC, Siegelman et al. showed in an analysis of behavioural data from the ELP that no effect of Marelli and Amenta’s (2018) token-based OSC was observed when controlling for this frequency–ratio between the target word and that of its neighbours. The authors used LSA semantic vectors as in Marelli et al. (2015), but they also showed that when they calculated OSC using global vectors (GloVe) for word representation (Pennington et al., 2014), OSC still predicted response latencies and accuracy in the lexical decision data. Both LSA and GloVe are count-based models in that they define vectors using co-occurrence matrices that encode information about how often words occur in the same linguistic context as the word of interest.

Siegelman, Rueckl, et al. (2022) further examined whether the effects of OSC can be observed in sentence reading using eye-tracking data extracted from the GECO book reading corpus (Cop et al., 2017). The authors found a significant effect of OSC on reading times such that shorter gaze durations were produced for words that had higher OSC than those that had lower OSC. They concluded that their findings provide evidence that proficient readers are sensitive to orthographic-semantic regularities in the language and rely more heavily on these regularities in reading tasks with greater semantic involvement.

Hendrix and Sun (2021) also showed that OSC predicted English lexical decision performance using yet another method for calculating semantic vectors and a different definition of orthographic neighbours. Hendrix and Sun used a prediction-based approach to generate semantic vectors, as did Marelli and Amenta (2018). Specifically, they used the fastText extension of a skip-gram model (Bojanowski et al., 2017). Hendrix and Sun argued that this approach produces better quality semantic vectors, particularly for words that appear infrequently in the input data, because it takes subword information into account. Semantic vectors are derived not only for a word but also for all component 3-grams to 6-grams, and then these vectors are summed. The orthographic neighbours of a word were defined as the five words with the shortest Levenshtein distance to the word. OSC was calculated as the average semantic similarity between a word and its five neighbours (a type-based measure), and then this value was log transformed. OSC was a significant predictor of lexical decision response times taken from the British Lexicon Project. Frequency, length, OLD20, bigram frequency, and semantic neighbourhood density were included in the model, although not root family size. OSC was also a significant predictor of lexical decision performance when the authors derived the semantic vectors for words and their neighbours using one of the prediction approaches used by Marelli and Amenta (word2vec skip-gram model), which was not surprising because the two OSC measures were highly correlated. Hendrix and Sun reported that a token-based version of their OSC measure was a substantially weaker predictor of lexical decision performance than a type-based measure.

In summary, effects of OSC on word recognition have been found using a range of methods to calculate values. Semantic vectors have been generated using count-based (LSA, GloVe) and prediction-based (word2vec skip-gram, word2vec CBOW, fastText skip-gram) models. It is not yet clear whether one of these is better than the others, but Hendrix and Sun (2021) claim that fastText produces higher quality semantic vectors for words that appear infrequently. This is a desirable quality if many of the orthographic neighbours of a word are low in frequency. The orthographic neighbourhood of a word has been defined in a variety of ways. Siegelman, Rueckl, et al. (2022) provide a convincing argument that it is best to avoid including the word itself in the neighbourhood, and Marelli and Amenta’s (2018) work suggests that defining neighbours as those that embed the word produces OSC values that are more predictive of reading times than definitions of neighbours based on Levenshtein distance. The works of Siegelman, Rueckl, et al. (2022) and Hendrix and Sun (2021) provide evidence that a type-based version of OSC is a better predictor of reading times than a token-based version, but it is worth exploring this issue further. And finally, of the four studies discussed, only Marelli and Amenta (2018) and Marelli et al. (2015) demonstrated that OSC predicts reading times when root family size is included in a model.

Siegelman, Rueckl, et al. (2022) noted that an important outstanding issue is whether effects of OSC are observed in other languages besides English that have a different mix of regularities between form and meaning. The goal of the present study is to explore the relationship between spelling and meaning in Malay via estimates of root family size and OSC and to provide behavioural evidence of their impact on Malay readers. In our calculations of OSC, semantic vectors generated using Bojanowski et al.’s (2017) fastText (skip-gram) method were employed. We considered the critiques made by Siegelman, Rueckl, et al. (2022) of Marelli et al.’s (2015) token-based OSC and included both type and token-based measures, neither of which included the word itself in its neighbourhood. Marelli and Amenta’s (2018) definition of orthographic neighbours was chosen (target plus additional letters). In an agglutinative language like Malay, Siegelman’s et al. operational definition of orthographic neighbours (a Levenshtein distance of one between word pairs) would leave out a considerable number of morphologically related words from the neighbourhood, specifically all morphologically related words with an affix longer than one letter. Similarly, Hendrix and Sun’s (2021) definition of neighbourhood as the closest five neighbours based on Levenshtein distance would also likely include too few of a Malay word’s morphological relatives.

### Malay language (*Bahasa Melayu*)

The Malay language, also known as *Bahasa Melayu*, is an Austronesian language spoken in various regions of Southeast Asia such as in Indonesia, Malaysia, Singapore, and Brunei. Malay is highly agglutinative, that is, morphemes are combined to create words and express grammatical relationships. Affixes are extensively used in Malay to form words that share related meanings (e.g., *sunyi*/*quiet; ke-sunyi* ***-*** *an*/*silence; makan*/*eat; makan*-*an*/*foods; cakap*/*talk; per-cakap-an*/*conversation*). While in these examples the English translations that share related meanings do not share a spelling pattern, the Malay equivalents do. Shorter words, however, tend to overlap in spelling with other words that do not share a meaning, for example, *aku/I; paku/nail (fastener); takut/afraid*. In addition, Malay has a relatively shallow orthography, that is, a consistent and predictable mapping between spelling and sound. In some cases of derived word relatives, the first letter of the root is dropped to facilitate pronunciation. For example, the derived word *mengutip* **/***to pick up* (*something small*) contains the root *kutip* in which the letter *k* is dropped. Note that although the primary focus in the present study is on monomorphemic words, word neighbours for each monomorphemic word are considered in the calculations of OSC.

### The present study

The present study consists of two parts. First, we further developed an existing large Malay word database, the MLP, by adding a measure that captures word meaning. The MLP, originally assembled by Yap et al. (2010), included a wide range of lexical properties such as frequency and length for 9,592 Malay words. A morphological version of the database, the MLP2, showed the morphological segmentation for each word and added a large set of distributional morphological properties (e.g., root and affix family size, morphemic length, affix productivity) for each word in the database (Maziyah Mohamed et al., 2023). Here our first goal is to supplement the MLP2 (creating MLP3) by computing the osc for a large set of monomorphemic words in the database. In addition, new lexical decision latencies were collected for 1,280 Malay words of various morphological structures in the MLP. Of most interest to the present analyses were decision latencies for monomorphemic words. The MLP3 can be accessed using the following link: https://osf.io/dhyzb/?view_only=e22e226ac62c4fd2b54de5d27467404e.

Our second and main goal was to investigate a series of questions concerning the impact of root family size and osc on decision latencies of monomorphemic words in Malay readers. We first established whether root family size and osc independently were significant predictors of decision latencies in monomorphemic words and explored their interactions with frequency. Next, we compared type and token-based calculations of osc as predictors of Malay decision latencies. The final set of models included both root family size and osc. One question that we explored was whether osc remains a significant predictor of decision latencies when frequency and root family size were controlled. A second question was whether there is an interaction between root family size and osc. With respect to the second question, Tyler et al. (2000) found a greater impact of imageability (i.e., a variable that measures some semantic information) in words with a large cohort size, that is, a large number of words that share the same onset. The authors explained that as cohort size increases, phonological information for each word becomes less unique. Consequently, listeners must rely on some other word information to discern between lexical items. These results are a hint that we might see a larger effect osc for words in large morphological families.

In summary, our study was designed to provide insights about the distributional properties of the language that readers of Malay pick up on, specifically whether they are sensitive to OSC. We expect that the data provided by our study will be useful in the future development and testing of Malay versions of computational models of word recognition. However, it is beyond the scope of the current investigation to develop such a model.

### MLP3: computing OSC

There are a few steps to calculating OSC. The first step was to identify the set of orthographic neighbours from the words in the MLP database for each monomorphemic word. As noted previously, we adopted Marelli and Amenta’s (2018) definition of orthographic neighbours. For example, *kelapa* (coconut) has neighbours *kelapan* (eighth), *kelapangan* (vastness), and *kelaparan* (hunger). In addition, in some derived words, we considered the change in the spelling of the root (e.g., *kutip—*pick up; *mengutip*—to pick up). In those cases, *mengutip* is a neighbour of *kutip.*

The second step was to obtain semantic vectors for each monomorphemic word and each of their neighbours. We first consulted Grave et al. (2018), who provided pre-rained word vectors using fastText (CBOW) for 157 languages, including Malay. However, only 2,400 Malay words from the MLP had semantic vectors in that source. We then found another resource from the fastText group, Wiki word vectors, that provided pretrained word vectors of 300-dimensions for 294 languages, including Malay (https://fasttext.cc/docs/en/pretrained-vectors.html). These vectors were generated using Bojanowski et al.’s (2017) fastText (skip-gram) method, and the data source for the vectors was Wikipedia. About 92% of the words in the MLP had vectors in this resource.

The next step in calculating OSC was to calculate the mean semantic similarity (i.e., cosine similarity) between the semantic vector for a target stem and the vectors for each of its orthographic neighbours. The assumption is that the higher the co-occurrence of a word pair in similar contexts, the greater the overlap there is in their meanings, and the smaller the angle (i.e., closer) will be between their semantic vectors. Token-based O-S consistency (Marelli & Amenta, 2018; Marelli et al., 2015) and type-based O-S consistency (Siegelman, Rueckl, et al., 2022) are expressed by the following formulas:

Token-based osc

$$\mathrm{OSC}\left(t\right)=\;\frac{\sum_{x=1}^{k}f_{r_{x}}.\;cos(\vec{t},\;\vec{r_{x}})}{\sum_{x=1}^{k}f_{r_{x}}}$$

Type-based osc

$$\mathrm{OSC}\left(t\right)=\;\frac{\sum_{x=1}^{k}cos(\vec{t},\;\vec{r_{x}})\;}{k}$$

In both formulas, *t* is the target word, and *r_x_* represents each orthographic neighbour of the target *t*. A difference between the two formulas is in the $f_{r_{x}}$ term in token-based OSC, and the *k* term in type-based OSC. $f_{r_{x}}$ refers to the corresponding frequencies of each orthographic neighbour, and *k* represents the neighbourhood size of target *t* (i.e., number of orthographic neighbours). Of the 4,094 monomorphemic words in the MLP, we calculated OSC estimates for the 2,287 words that had a vector in the Wiki word vector resource and had at least one neighbour. Estimates of OSC range from 0 to 1, where values closer to 1 suggest a greater consistency with which spelling patterns map onto meanings.

In the present study, we computed both type-based osc using the formula provided in Siegelman, Rueckl, et al. (2022), and token-based, Rueckl, such as in Marelli and Amenta (2018), except that the target word was excluded from its neighbourhood. An objective was to explore whether one of these OSC measures is a significant predictor of lexical decision latencies for monomorphemic Malay words.

## Method

### Participants

A total of 141 native Malay speakers (*M*_age_ = 34.5 years) were recruited via Cloud Research. Participants were compensated with the amount they had agreed upon with the platform through which they entered the study. Participants’ self-ratings of their proficiency levels in Standard Malay and English are reported in Table 1. Data were excluded from an additional 45 participants who obtained an accuracy rate lower than 75% on the lexical decision task.

**Table 1.** Self-ratings of language proficiency.

	Malay		English	
	*M*	*SD*	*M*	*SD*
Understanding	9.21	1.06	8.56	1.25
Speaking	9.09	1.27	8.13	1.45
Reading	9.47	0.95	8.66	1.32
Writing	9.03	1.36	8.19	1.50

*Note.* Range is from 1 (not fluent) to 10 (very fluent).

### Stimuli

From the 2,287 monomorphemic words that had osc scores, we selected 1,000 words that did not already have lexical decision latencies in MLP2. These were the critical words in the present study. To vary the morphological structure of the word stimuli in the experiment, we selected an additional 280 multimorphemic filler words of a variety of morphological structures that did not already have lexical decision latencies in the MLP. In addition, 1,000 pseudowords without an affix and 280 pseudowords with one or more legal affixes were included for the purpose of a lexical decision task. All had nonword roots. Of these, 1,033 were extracted from previous studies (Maziyah Mohamed et al., 2023; Yap et al., 2010), and the remaining 247 pseudowords were created by a native Malay speaker. We carefully ensured that the structure of the pseudowords and their corresponding ratio resembled the set of real words. All pseudowords were checked against a Malay dictionary, *Kamus Dewan*, to ensure they were indeed not real words. A complete list of 3,047 pseudowords from the present study and previous abovementioned studies of Malay are available in the URL above. The stimuli were divided among four lists with each list consisting of 320 unique words, and 320 unique pseudowords. In all, 45 participants completed the first list, and 32 participants each completed the remaining lists.

### Procedure

Participants were first screened on their knowledge of Malay in a short pre-test. In the pre-test, participants were presented with 5 Malay words and 5 nonwords, and they were asked to decide whether each of the 10 letter strings is a Malay word or a nonword. Participants who obtained a score of 8 or more out of 10 were invited to complete the lexical decision task. The lexical decision task was programmed using PsychoPy version 2022.1.4 (Peirce et al., 2019) and was hosted online on Pavlovia. In the lexical decision task, participants were instructed to decide if the letter string presented on the screen was a word or a nonword in Malay, and to press one of two keys (i.e., 0 or 1) as quickly and as accurately as possible. The lexical decision task was completed in a single session. Each session consisted of eight blocks, with each block containing approximately 80 trials. Blocks, and trials within each block, were randomised. Letter strings were presented in white, and lowercase Courier letters with a height of .05 unit (one unit corresponds to the maximum height of the computer screen when the device is positioned in landscape). On each trial, a fixation cross “+” appeared on the centre of the screen for 500 ms, followed by a letter string that remained on the screen until the participant responded or for a maximum of 3,000 ms. After each response, participants were provided with feedback on whether their response was correct, wrong, or that no response was recorded. The feedback was presented during a 750 ms intertrial stimulus interval. The purpose of the feedback was to help maintain the participant’s attention on the task. At the end of each block, participants were given a break and were instructed to press “SPACE” to resume. At the end of the lexical decision task, participants were automatically redirected to a language background questionnaire on Qualtrics. Participants were asked to rate their ability to understand, speak, read, and write in Malay and English. The study was conducted entirely in Malay and lasted approximately 30 min.

## Results

### Overview of analyses

The overall accuracy rate on the 2,560 stimuli was 90.1% (88.4% for words and 91.8% for nonwords). Of the 1,000 monomorphemic words that had decision latencies, we excluded 92 words that had fewer than 80% of their orthographic neighbours assigned a vector from the Wiki word vectors resource. These 908 words were crucial to the analyses of the present study. Incorrect responses (11.8%) and response times (RTs) that were shorter than 350 ms or longer than 3,000 ms were excluded. In addition, only response times that were 2.5 *SD*s within each participant’s overall mean were retained. There was no speed accuracy trade-off on these 908 critical words—words that had a higher accuracy tended to have faster (i.e., lower) response times (*r* = −.64). All remaining response times were inverse transformed so that residuals are approximately normal. These trial-level data can be obtained from the URL above.

To examine the impact of root family size and osc on lexical decision latencies and their interaction with frequency, we conducted a series of generalised additive mixed models (GAMMs) in R (R Core Team, 2023). Below we report our procedure for each GAMM with careful considerations of concurvity to ensure that each model is interpretable.

Each model was run using the “*bam”* function and the “*discrete* *=* *TRUE”* argument (Li & Wood, 2019; Wood et al., 2017) from the “mgcv” package (Wood, 2017). Both function “*bam*” and “*discrete* *=* *TRUE*” make fitting a GAMM to a large dataset much faster. Where necessary, the number of basis functions *k* (where *k* is the maximum possible degrees of freedom allowed for a smooth term and has a default value of 10) was reduced to five. In each model, whole-word frequency was included. Another lexical variable typically accounted for is word length. In the present study, length is moderately correlated with O-S consistency (type-based, *r* = .57; token-based, *r* *=* .58). Shorter words score lower on osc likely because such words (e.g., *akar*—root) tend to share an orthographic overlap with other words that do not share similar meanings (e.g., *bakar*—burn; *kelakar*—funny). We did not include length in the models presented here because the concurvity statistics for each model substantially increased when length was entered as a predictor. An orthographic familiarity variable (e.g., OLD20) is also sometimes included in models as a control variable. We did not include it here because it had a very low correlation with inverse RT (*r* = .015). However, we did also run our models with OLD20. Including this variable made little difference to the results for OSC and the overall fit of the models was never improved (see also Hendrix & Sun, 2021). Random effects in each model included trial number and subjects, using a random factor smooth interaction (i.e., *b*s = “*f*s”), and a directive *m* = 1 specified. This structure of random effects was adopted from a GAMM tutorial (see Chuang et al., 2020). By using a factor smooth in the random effects structure, we modelled the nonlinear equivalent in a GAMM of what is typically modelled in a linear mixed model that has by-subject random intercepts and random slopes for trials. The directive *m* = 1 is specified so that a factor smooth is allowed to eliminate linear trends when there is no evidence to support such trends. In such a case, the factor smooth returns horizontal lines with different intercepts (i.e., random intercepts). All diagnostic plots of model residuals and concurvity statistics are available in the Supplementary Materials. The correlation matrix and descriptive statistics of each variable of interest is presented in Tables 2 and 3, respectively.

**Table 2.** Correlation matrix of variables for 908 monomorphemic words.

	1.	2.	3.	4.
1. Frequency				
2. Length	−.052			
3. Root family size	.200***	.205***		
4. OSC (Token)	−.198***	.598***	.238***	
5. OSC (Type)	−.213***	.604***	.240***	.990***

OSC: orthographic–semantic consistency.

*Note.* Word frequency and family size were log transformed.

*** *p*< .001.

**Table 3.** Descriptive statistics of variables for 908 monomorphemic words.

	*M*	Median	*SD*	Minimum	Maximum
Frequency	1.39	1.32	.878	.057	4.15
Length	5.07	5.00	1.07	2.00	10.0
Root family size	.538	.477	.173	.301	1.00
OSC (Token)	.534	.551	.167	.132	.951
OSC (Type)	.537	.557	.163	.136	.951

OSC: orthographic–semantic consistency.

*Note.* Word frequency and family size were log transformed.

### GAMM

Three GAMMs were run, each with one variable of interest (i.e., root family size, type-based osc, and token-based osc) to first establish whether root family size and osc are significant predictors of decision latencies in Malay monomorphemic words. Then, a variation of each of these models was run to explore the interaction of the variable of interest with frequency. We compared the models to determine whether type-based or token-based osc was a better predictor of decision latencies in Malay readers, and evaluated whether the model that had either root family size or osc better accounted for our data. The final set of models included both root family size and osc. We explored whether osc remains a significant predictor of decision latencies when root family size is in the model, and whether there is an interaction between root family size and osc.

Recall that the critical difference between osc and root family size is in the word relatives that are captured by each of these measures. If the model that accounts for osc is a better-fitting model than the model that accounts for root family size, then it would suggest that orthographic neighbours that do not share meaning have a negative impact on Malay reading speed. Conversely, if the model that accounts for root family size is a better-fitting model than the model that accounts for osc, then one interpretation is that the orthographic neighbours that do not share meaning have little impact on Malay readers.

#### Root family size

In studies of Malay as previously noted, root family size was a significant predictor of decision latencies for Malay words that had one prefix, one root, and one suffix (Maziyah Mohamed et al., 2023), as well as in words with one prefix and one root (Maziyah Mohamed & Jared, 2024). Similarly, for monomorphemic words, we expected that root family size would significantly impact decision latencies. Whole-word frequency and root family size were log transformed and entered as predictors in the model. An *s* thin plate regression spline smooth was used for frequency and root family size. Random effects included subjects and trials, as previously explained in detail. The syntax for this model is:

$$\begin{array}{l} inverse\;RT\;∼\;s(frequency)\;+\;s(root\;family\;size)\\ +\;list\;+\;s(trial\;number,\;subject,\;bs\;=\;\prime fs\prime ,\;m\;=\;1),\;\;data. \end{array}$$

**Figure 1.**
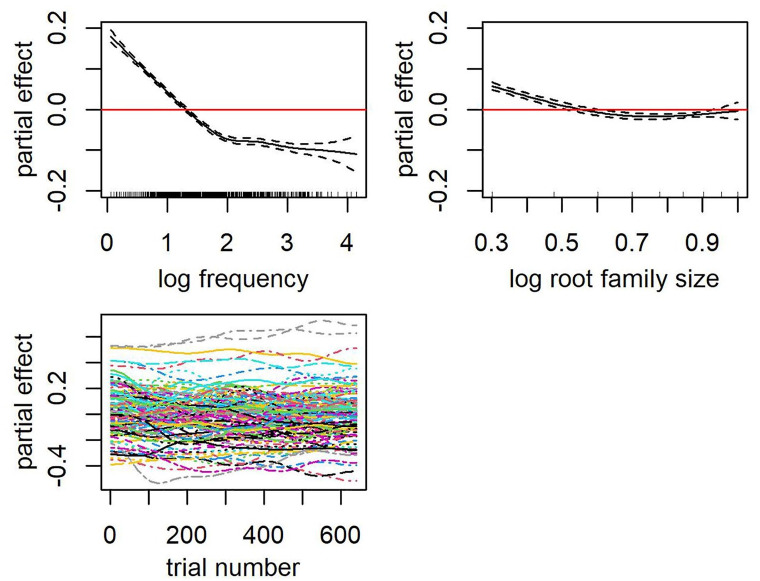
Top row: Partial effects of frequency (left) and root family size (right). Rugged lines on the *x*-axes of each partial plot represent the distribution of the data. Bottom row: Partial effects of trial number by subjects (left).

The first two terms after the tilde account for the main effect of frequency and root family size, respectively. Of particular interest, root family size was a significant predictor of RT when whole-word frequency was controlled (see Table 4). A facilitative effect of root family size was observed, that is, the larger the root family size the faster the RT (see partial effect plot in Figure 1). The impact of root family size on decision latencies is greater with each increment for words that have smaller root families than those with larger root families. This effect of root family size on decision latencies observed in monomorphemic words mirrored that in Malay words of other morphological structures in previous studies of Malay.

**Table 4.** Model output; Root family size.

Parametric coefficients		Estimate	SE	*t*	*p*
	Intercept	−1.43	.02	−77.48	<.0001
	List two	.04	.01	4.46	<.0001
	List three	.02	.01	2.87	.004
	List four	−.01	.01	−1.55	.122
Approximate significance of smooth terms		e*df*	Ref. *df*	*F*	*p*
	Frequency	6.01	7.20	347.73	<.0001
	Root family size	3.08	3.50	75.77	<.0001
	Trial number, subjects	562.60	1,034.00	13.90	<.0001
*R*^2^ (adj.)	.39				
AIC	4,855.91				

AIC: Akaike information criterion.

*Note.* Word frequency and root family size were log transformed. The model syntax is inverse *RT* ~ *s*(frequency) + *s*(root family size) + *list* + *s*(trial number, subjects, *bs* = “*fs*”, *m* = 1), data.

Next, we ran a variation of the model above by adding an interaction between frequency and root family size. The two previous studies of Malay, mentioned above, found a significant interaction between frequency and root family size on decision latencies in morphologically complex words. Specifically, those studies observed a facilitatory effect of root family size that was particularly evident in lower frequency words. In the present study, we investigated whether the interaction between frequency and root family size on decision latencies exhibits a similar pattern for monomorphemic words. To examine the interaction between frequency and root family size, a *te* tensor product smooth was used. The syntax for this model is:

$$\begin{array}{l} inverse\;RT\;∼\;te(frequency,\;root\;family\;size)\;\\ +\;list\;+\;s(trial\;number,\;subject,\;bs\;=\;\prime fs\prime ,\;m\;=\;1),\;data. \end{array}$$

**Figure 2.**
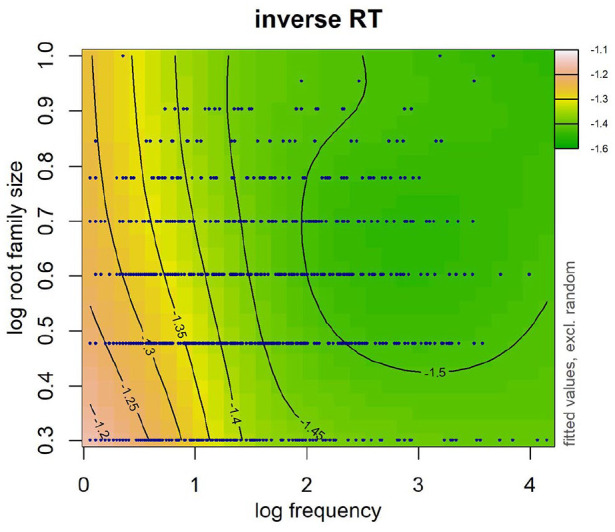
Word frequency and root family size were log transformed. Data are represented by dark blue points. Warmer colours (e.g., pink, orange) on the left-hand side denote longer RTs and cooler colours (e.g., green) on the right-hand side denote shorter RTs. Numbers on contour lines represent fitted inverse RT values.

Of most interest is the first term after the tilde that accounts for the main effect of frequency and root family size as well as their interaction. A significant interaction between frequency and root family size was observed (see Table 5). In particular, a facilitative effect of root family size was observed in lower frequency words (see Figure 2). No effect of root family size was observed for high-frequency words. The model that included the interaction between frequency and root family size fit the data better than the model without the interaction (∆AIC = 14.5).

**Table 5.** Model output; Root family size by frequency.

Parametric coefficients		Estimate	*SE*	*t*	*p*
	Intercept	−1.43	.02	−77.69	<.0001
	List two	.04	.01	4.47	<.0001
	List three	.02	.01	2.80	.005
	List four	−.01	.01	−1.54	.124
Approximate significance of smooth terms		*edf*	Ref. *df*	*F*	*p*
	Frequency, Root family size	13.11	15.40	196.21	<.0001
	Trial number, subjects	563.33	1,034.00	13.91	<.0001
*R*^2^ (adj.)	.39				
AIC	4,841.37				

AIC: Akaike information criterion.

*Note.* Word frequency and root family size were log transformed. The model syntax is inverse *RT* ~ *te*(frequency, root family size) + *list* + *s*(trial number, subjects, *b*s = “*f*s”, *m* = 1), data.

#### Orthographic-semantic consistency

In two additional models, we entered frequency and OSC as predictors in the model. Token-based and type-based OSCs were entered in separate models. Our goal was to determine whether OSC was a significant predictor of decision latencies, and to evaluate whether token-based or type-based OSC was a better predictor of decision latencies. Whole-word frequency was log transformed in both models. In both models, an *s* thin plate regression spline smooth was used for frequency and OSC. The structure of random effects was identical to the models above. The syntax for these models is:

$$\begin{array}{l} inverse\;RT\;∼\;s(frequency)\;+\;s(OSC)\;+\;list\;\\ +\;s(trial\;number,\;subject,\;bs\;=\;\prime fs\prime ,\;m\;=\;1),\;\;data. \end{array}$$

**Figure 3.**
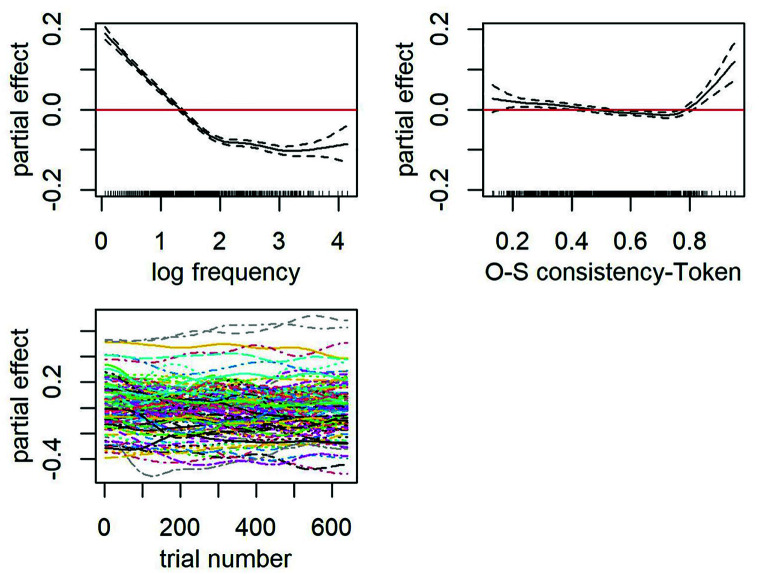
Top row: Partial effects of frequency (left) and OSC-Token (right). Rugged lines on the *x*-axes of each partial effect plot represent the distribution of the data. Bottom row: Partial effects of trial number by subjects (left).

**Figure 4.**
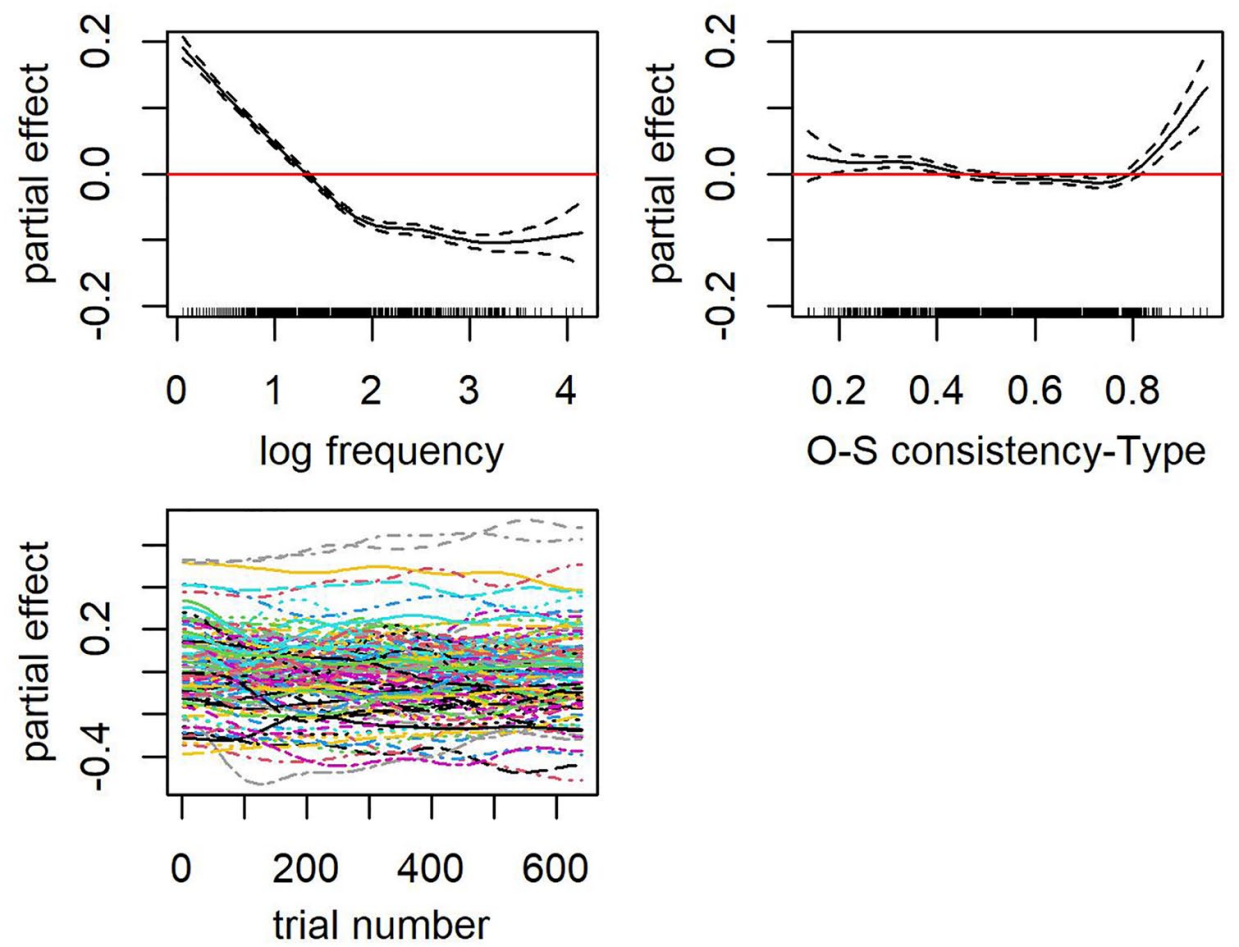
Top row: Partial effects of frequency (left) and OSC-Type (right). Rugged lines on the *x*-axes of each partial effect plot represent the distribution of the data. Bottom row: Partial effects of trial number by subjects (left).

Of interest in the present study is the second term after the tilde that accounts for the main effect of OSC. Both token-based (see Table 6) and type-based OSC (see Table 7) were significant predictors of decision latencies in Malay monomorphemic words when whole-word frequency was controlled. A facilitative effect of OSC was observed for most words across a wide range of OSC estimates, that is, the greater the consistency with which spelling patterns map onto meaning the faster the decision latencies (see partial effect plot in Figure 3 for token OSC and Figure 4 for type OSC). Both partial effect plots of OSC revealed a greater impact on decision latencies with each increment for words on the lower end of OSC (.1 ⩾ OSC < .5) than those that are more consistent (.5 ⩾ OSC < .8). Somewhat unexpected is the inhibitory effect of OSC for words that are highly consistent in their mapping of spelling to meaning (⩾.8), in which greater OSC scores for these words elicited slower RTs. Note, however, that there is only a small number of words that obtained a high estimate of token-based OSC (*n* = 41) and type-based OSC (*n* = 39), and most of these had a single orthographic neighbour. OSC is perhaps less representative in these words because the extent to which spelling patterns map onto meaning for each word is solely estimated by one other word relative. Crucially, we excluded these words in subsequent models. Both token and type OSC accounted for a similar amount of variance. Type-based OSC, however, emerged as a slightly better predictor than token OSC. The model that included a type-based OSC produced a slightly lower AIC than the model that included the token-based OSC (∆AIC = 5.7).

**Table 6.** Model output; orthographic-semantic consistency (Token).

Parametric coefficients		Estimate	SE	*t*	*p*
	Intercept	−1.42	.02	−77.82	<.0001
	List two	.04	.01	4.53	<.0001
	List three	.02	.01	2.91	.003
	List four	−.02	.01	−1.63	.10
Approximate significance of smooth terms		*edf*	Ref. *df*	*F*	*p*
	Frequency	6.26	7.43	335.01	<.0001
	OSC (Token)	6.17	7.38	8.45	<.0001
	Trial number, subjects	561.96	1,034.00	13.81	<.0001
*R*^2^ (adj.)	.385				
AIC	5,061.19				

OSC: orthographic–semantic consistency; AIC: Akaike information criterion.

*Note.* Word frequency was log transformed. The model syntax is inverse *RT* ~ *s*(frequency) + *s*(OSC) + *list* + *s*(trial number, subjects, *bs* = “*fs*”, *m* = 1), data. OSC (Token) = OSC (Token).

**Table 7.** Model output; orthographic-semantic (Type).

Parametric coefficients		Estimate	SE	*t*	*p*
	Intercept	−1.41	.02	−77.84	<.0001
	List two	.04	.01	4.58	<.0001
	List three	.02	.01	2.94	.003
	List four	−.01	.01	−1.60	.11
Approximate significance of smooth terms		*edf*	Ref. *df*	*F*	*p*
	Frequency	6.25	7.42	336.40	<.0001
	OSC (Type)	6.57	7.74	8.81	<.0001
	Trial number, subjects	561.82	1,034.00	13.81	<.0001
*R*^2^ (adj.)	.386				
AIC	5,055.48				

OSC: orthographic–semantic consistency; AIC: Akaike information criterion.

*Note.* Word frequency was log transformed. The model syntax is inverse *RT* ~ *s*(frequency) + *s*(OSC) + *list* + *s*(trial number, subjects, *bs* = “*fs*”, *m* = 1), data. OSC (Type) = OSC (Type).

Next, we explored whether there was a significant interaction between frequency and OSC. Again, separate models were run for token and type-based OSC. An interaction between frequency and OSC was added to the model using a *te* tensor product smooth. The structure of the random effects is identical to the models above. The syntax for these models is:

$$\begin{array}{l} inverse\;RT\;∼\;te(frequency,OSC)\;+\;list\;\\ +\;s(trial\;number,\;subject,\;bs\;=\;\prime fs\prime ,\;m\;=\;1),\;\;data. \end{array}$$

**Figure 5.**
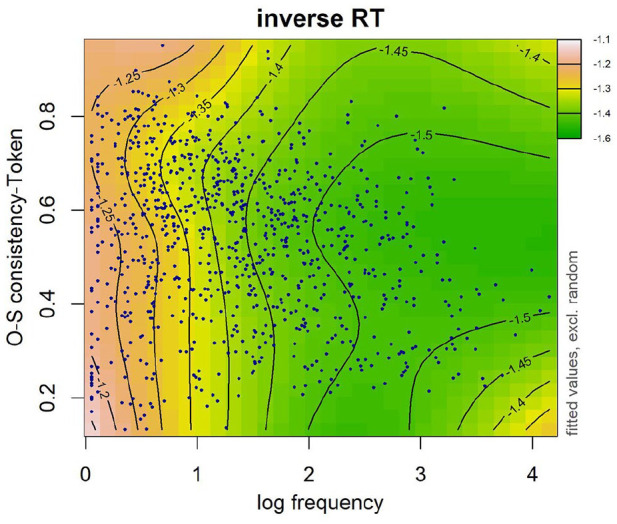
Word frequency was log transformed. Data are represented by dark blue points. Warmer colours (e.g., pink, orange) on the left-hand side denote longer RTs and cooler colours (e.g., green) on the right-hand side denote shorter RTs. Numbers on contour lines represent fitted inverse RT values.

**Figure 6.**
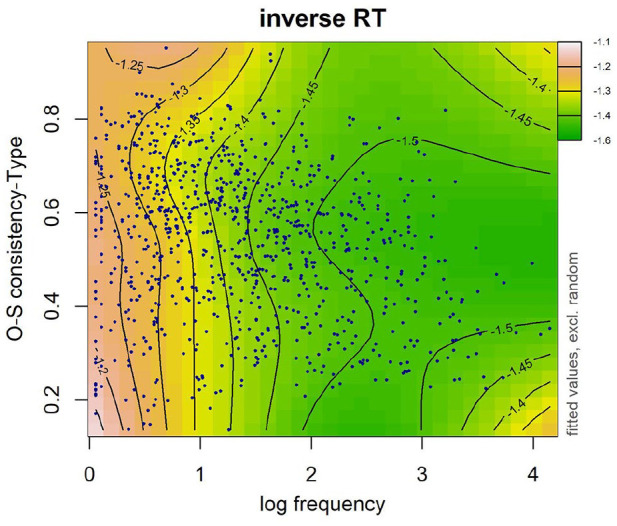
Word frequency was log transformed. Data are represented by dark blue points. Warmer colours (e.g., pink, orange) on the left-hand side denote longer RTs and cooler colours (e.g., green) on the right-hand side denote shorter RTs. Numbers on contour lines represent fitted inverse RT values.

The first term after the tilde accounts for the main effect of frequency and OSC as well as their interaction. A significant interaction between frequency and OSC was found in both token OSC (see Table 8) and type OSC (see Table 9). In particular, the effect of OSC described above was most evident in lower frequency words (see Figures 5 and 6). No effect of OSC was observed in high-frequency words. The models that included the interaction between frequency and either token-based OSC (∆AIC = 28.62) or type-based OSC (∆AIC = 25.89) were better-fitting models than the models that did not include this interaction.

**Table 8.** Model output; OSC (Token) by frequency.

Parametric coefficients		Estimate	SE	*t*	*p*
	Intercept	−1.41	.02	−77.65	<.0001
	List two	.04	.01	4.40	<.0001
	List three	.02	.01	2.71	.007
	List four	−.02	.01	−1.73	.08
Approximate significance of smooth terms		*edf*	Ref. *df*	*F*	*p*
	Frequency, OSC (Token)	18.21	20.55	137.10	<.0001
	Trial number, subjects	563.30	1,034.00	13.83	<.0001
*R*^2^ (adj.)	.386				
AIC	5,032.57				

OSC: orthographic–semantic consistency; AIC: Akaike information criterion.

*Note.* Word frequency was log transformed. The model syntax is inverse *RT* ~ *te*(frequency, OSC) + *list* + *s*(trial number, subjects, *bs* = “*f*s”, *m* = 1), data. OSC (Token) = Orthographic-semantic consistency (Token).

**Table 9.** Model output; OSC (Type) by frequency.

Parametric coefficients		Estimate	SE	*t*	*p*
	Intercept	−1.41	.02	−77.64	<.0001
	List two	.04	.01	4.41	<.0001
	List three	.02	.01	2.71	.006
	List four	−.02	.01	−1.74	.08
Approximate significance of smooth terms		*edf*	Ref. *df*	*F*	*p*
	Frequency, OSC (Type)	18.37	20.66	136.51	<.0001
	Trial number, subjects	563.02	1,034.00	13.83	<.0001
*R*^2^ (adj.)	.386				
AIC	5,029.59				

OSC: orthographic–semantic consistency; AIC: Akaike information criterion.

*Note.* Word frequency was log transformed. The model syntax is inverse *RT* ~ *te*(frequency, OSC) + *list* + *s*(trial number, subjects, *bs* = “*fs*”, *m* = 1), data. OSC (Type) = Orthographic-semantic consistency (Type).

#### Comparing models of root family size and orthographic-semantic consistency

In sum, the models that included the interaction between frequency and each variable of interest (i.e., root family size, token-based OSC, and type-based OSC) better accounted for our data than each version of those models that did not include the interaction. Across all models presented, the model that included root family size and its interaction with frequency produced a substantially better model fit to the data (AIC = 4,841.37) than the model equivalent of either token-based OSC (AIC = 5,032.57) or type-based OSC (AIC = 5,029.59). Recall that a notable difference between root family size and OSC is in the types of neighbours that are captured in each of these measures. Root family size represents the number of morphological neighbours of a particular word (i.e., words that share an overlap in spelling pattern and meaning). OSC estimates the extent to which spelling patterns map onto meaning and therefore, in contrast to root family size, includes both morphological neighbours and purely orthographic neighbours (i.e., words that are spelled similarly but differ in their meaning) in its calculations. This finding suggests that purely orthographic word neighbours, that are captured by OSC but not root family size, may not have a large impact on Malay readers.

#### Is orthographic-semantic consistency a significant predictor when root family size is accounted for?

In the analyses just reported, we explored in separate models whether root family size and OSC were significant predictors of Malay decision latencies. The best-fitting model was the model that included the main effect of frequency and root family size as well as their interaction. Following the best-fitting model from the analyses above, here we added OSC as a predictor in the same model to examine whether OSC remains a significant predictor of Malay decision latencies when root family size is accounted for. To do so, an *s* thin plate regression spline smooth was used for OSC. Token-based and type-based OSC were entered in separate models. The syntax for each model is:

$$\begin{array}{l} inverse\;RT\;∼\;te(frequency,root\;family\;size)\;\;+\;s(OSC)\\ +list+s(trial\;number,\;subject,\;bs\;=\;\prime fs\prime ,\;m\;=\;1),\;data. \end{array}$$

**Figure 7.**
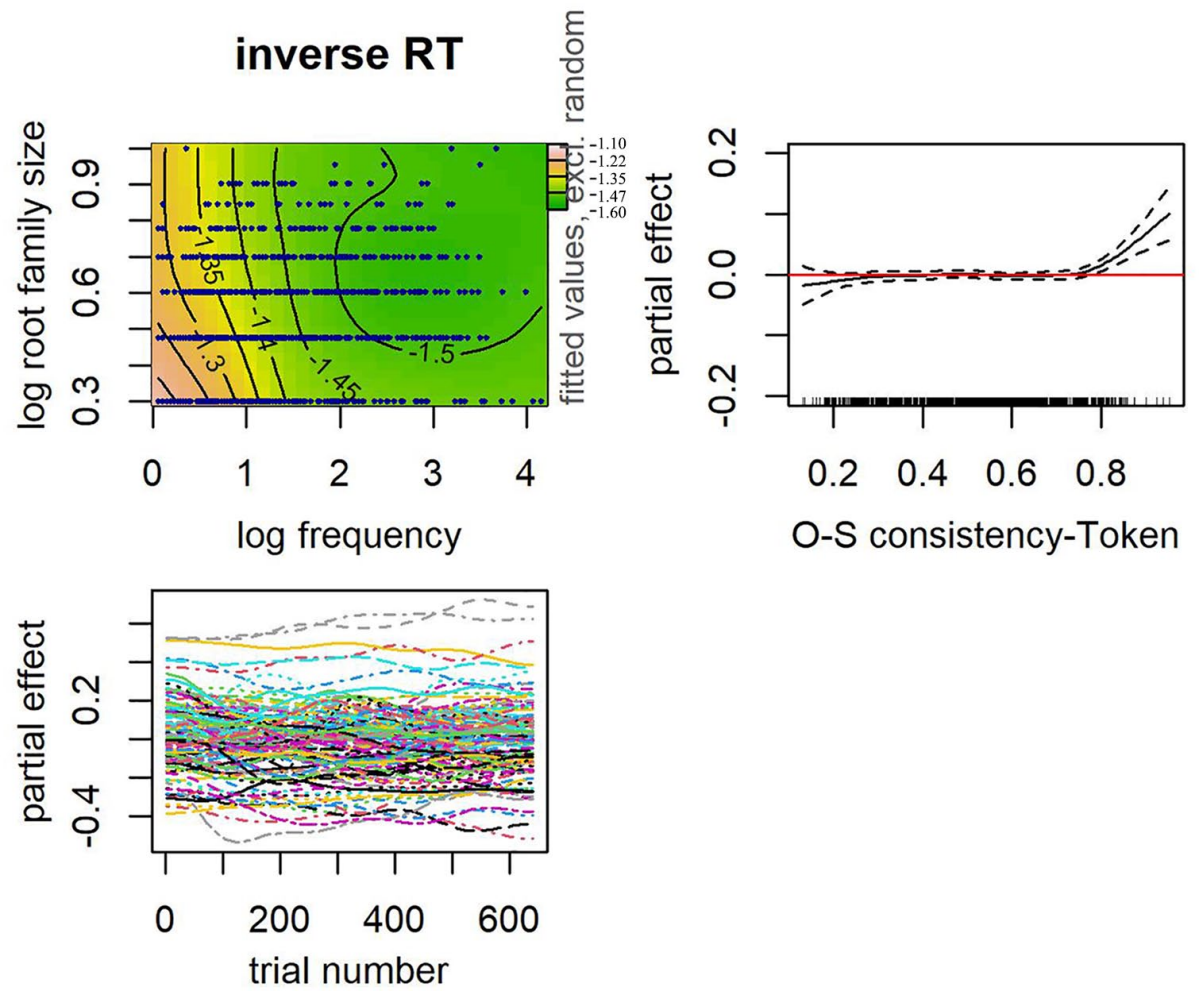
Top row: Interaction between frequency and root family size (left). Data represented by dark blue points. Warmer colours (e.g., pink, orange) on the left-hand side denote longer RTs and cooler colours (e.g., green) on the right-hand side denote shorter RTs. Numbers on contour lines represent fitted inverse RT values. Partial effect of orthographic-semantic consistency-Token (right), with rugged lines on the *x*-axis representing the distribution of the data. Bottom row: Partial effects of trial number by subjects (left).

**Figure 8.**
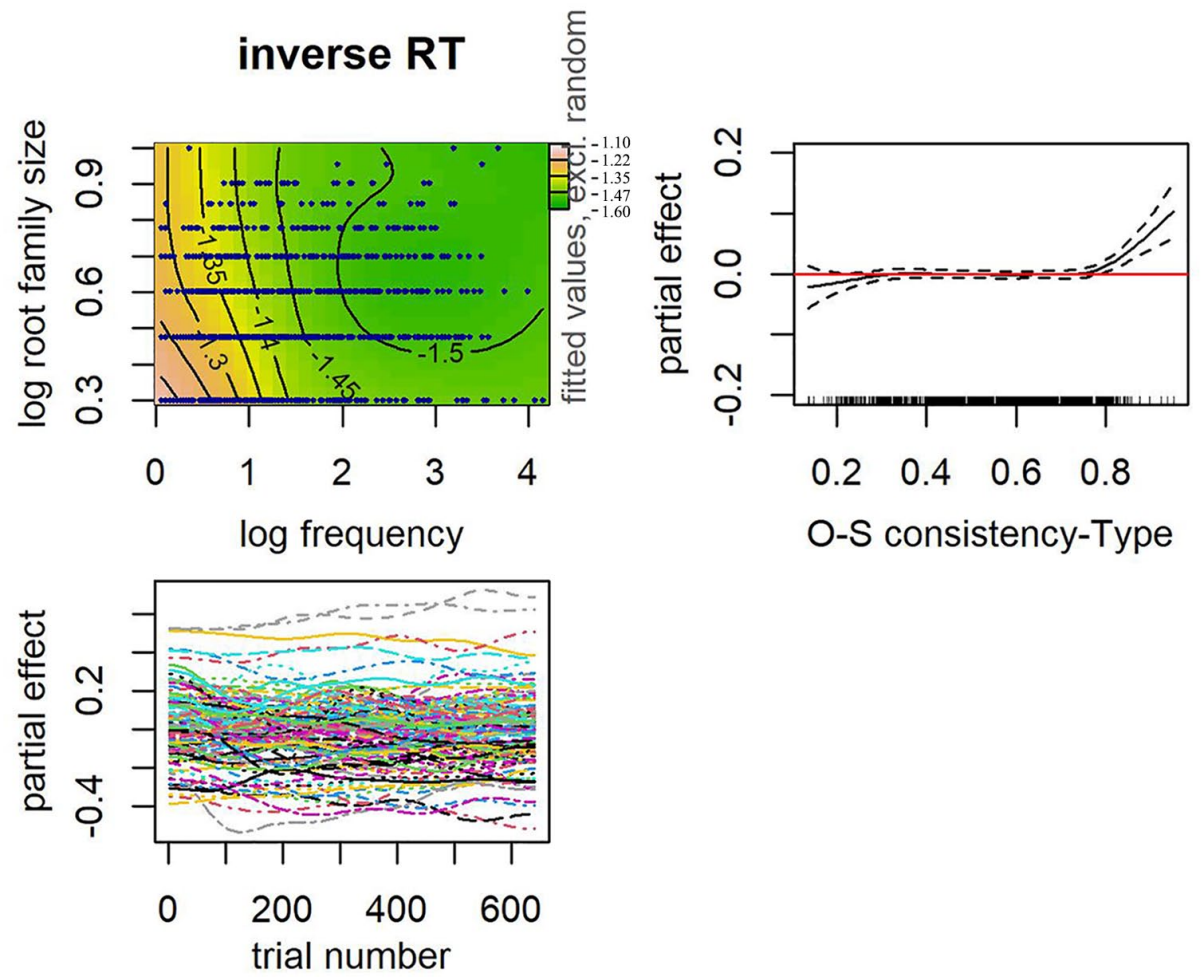
Top row: Interaction between frequency and root family size (left). Data represented by dark blue points. Warmer colours (e.g., pink, orange) on the left-hand side denote longer RTs and cooler colours (e.g., green) on the right-hand side denote shorter RTs. Numbers on contour lines represent fitted inverse RT values. Partial effect of orthographic-semantic consistency-Type (right) with rugged lines on the *x*-axis representing the distribution of the data. Bottom row: Partial effects of trial number by subjects (left).

In both models, the interaction between frequency and root family size was significant. Of particular interest is the second term after the tilde that accounts for the main effect of OSC. OSC remained a significant predictor of Malay decision latencies when root family size was entered in the same model, for both the type and token measure of OSC (see Tables 10 and 11). Compared with the model with root family size and its interaction with frequency (AIC = 4,841.37), models that also included OSC were a better fit, both for token-based OSC (AIC = 4,818.28) and type-based OSC (AIC = 4,820.93). Note, however, that the impact of increasing OSC on decision latencies is only apparent in words that are already highly consistent (see partial effect plots of OSC in Figure 7 for token-based and Figure 8 for type-based). Recall that words that are highly consistent (OSC ⩾ .8) in our data are represented by a small set of words that typically had only a single neighbour. Critically, in an additional set of analyses, we excluded the words that are highly consistent (39 words for type-based OSC, and 41 words for token-based OSC) and ran the same model, each for token and type OSC. In these additional analyses, token and type OSC were no longer a significant predictor of Malay decision latencies when root family size was included (see Tables 12 and 13). This finding further suggests that word relatives that share spelling patterns but carry a different meaning have little impact Malay readers.

**Table 10.** Model output; OSC (Token) with root family size by frequency.

Parametric coefficients		Estimate	SE	*t*	*p*
	Intercept	−1.43	.02	−77.36	<.0001
	List two	.04	.01	4.34	<.0001
	List three	.02	.01	2.68	.007
	List four	−.02	.01	−1.69	.090
Approximate significance of smooth terms		*edf*	Ref. *df*	*F*	*p*
	Frequency, Root family size	13.63	15.94	173.94	<.0001
	OSC (Token)	5.52	6.72	4.02	.0002
	Trial number, subjects	564.12	1,034.00	13.93	<.0001
*R*^2^ (adj.)	.391				
AIC	4,818.23				

OSC: orthographic–semantic consistency; AIC: Akaike information criterion.

*Note.* Word frequency and root family size were log transformed. The model syntax is inverse *RT* ~ *te*(frequency, root family size) + *s*(OSC Token) + *list* + *s*(trial number, subjects, *bs* = “*fs*”, *m* = 1), data. OSC (Token) = Orthographic-semantic consistency (Token).

**Table 11.** Model output; OSC (Type) with root family size by frequency.

Parametric coefficients		Estimate	SE	*t*	*p*
	Intercept	−1.43	.02	−77.39	<.0001
	List two	.04	.01	4.37	<.0001
	List three	.02	.01	2.70	.007
	List four	−.02	.01	−1.65	.099
Approximate significance of smooth terms		*edf*	Ref. *df*	*F*	*p*
	Frequency, Root family size	13.53	15.85	175.04	<.0001
	OSC (Type)	5.66	6.87	3.69	.0004
	Trial number, subjects	563.87	1,034.00	13.93	<.0001
*R*^2^ (adj.)	.391				
AIC	4,820.93				

OSC: orthographic–semantic consistency; AIC: Akaike information criterion.

*Note.* Word frequency and root family size were log transformed. The model syntax is inverse *RT* ~ *te*(frequency, root family size) + s(OSC-Type) + *list* + s(trial number, subjects, *bs* = “*fs*”, *m* = 1), data. OSC (Type) = Orthographic-semantic consistency (Type).

**Table 12.** Model output; OSC (Token) with root family size by frequency - OSC < .8).

Parametric coefficients		Estimate	SE	*t*	*p*
	Intercept	−1.43	.02	−77.31	<.0001
	List two	.04	.01	4.30	<.0001
	List three	.03	.01	3.07	.002
	List four	−.02	.01	−1.61	.107
Approximate significance of smooth terms		*edf*	Ref. *df*	*F*	*p*
	Frequency, Root family size	13.20	15.56	177.16	<.0001
	OSC (Token)	1.39	1.68	.31	.771
	Trial number, subjects	552.23	1,034.00	13.36	<.0001
*R*^2^ (adj.)	.389				
AIC	4,680.52				

OSC: orthographic–semantic consistency; AIC: Akaike information criterion.

*Note.* Word frequency and root family size were log transformed. The model syntax is inverse *RT* ~ *te*(frequency, root family size) + *s*(O-S consistency-Token) + *list* + *s*(trial number, subjects, *b*s = “*f*s”, *m* = 1), data. OSC (Token) = orthographic-semantic consistency (Token).

**Table 13.** Model output; OSC (Type) with root family size by frequency - OSC < .8).

Parametric coefficients		Estimate	SE	*t*	*p*
	Intercept	−1.43	.02	−77.50	<.0001
	List two	.04	.01	4.28	<.0001
	List three	.03	.01	3.00	.003
	List four	−.02	.01	−1.60	.109
Approximate significance of smooth terms		−*edf*	Ref. *df*	*F*	*P*
	Frequency, Root family size	13.25	15.59	175.37	<.0001
	OSC (Type)	1.81	2.27	.576	.574
	Trial number, subjects	554.45	1,034.00	13.40	<.0001
*R*^2^ (adj.)	.39				
AIC	4,671.30				

OSC: orthographic–semantic consistency; AIC: Akaike information criterion. *Note.* Word frequency and root family size were log transformed. The model syntax is inverse *RT* ~ *te*(frequency, root family size) + *s*(OSC-Type) + *list* + *s*(trial number, subjects, *bs* = “*fs*”, *m* = 1), data. OSC (Type) = orthographic-semantic consistency (Type).

Finally, we explored the interaction between root family size and OSC. Specifically, we investigated whether there is a more fine-grained impact of Orthographic-semantic on decision latencies only in words with large morphological families (large number of words that share the same root). Separate models were run for token and type Orthographic-semantic using the dataset without words having a consistency ⩾ .8. A variation of the model above was conducted where we switched the places of frequency and Orthographic-semantic in the model syntax. The syntax for each model is:

$$\begin{array}{l} inverse\;RT\;∼\;te(root\;family\;size,Orthographic-semantic)\\ +s(frequency)+list\\ +s(trial\;number,\;subject,\;bs\;=\;\prime fs\prime ,\;m\;=\;1),\;data. \end{array}$$

**Figure 9.**
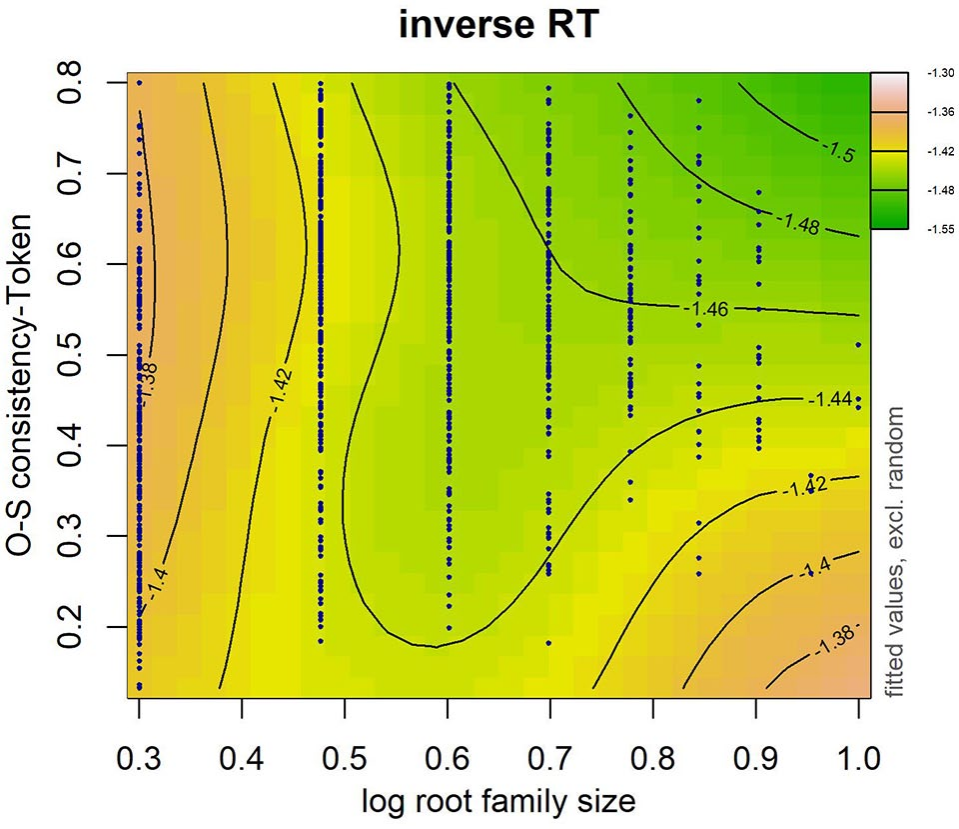
Word frequency was log transformed. Data are represented by dark blue points. Warmer colours (e.g., orange) on the left-hand side and the bottom right-hand side denote longer RTs and cooler colours (e.g., green) on the top half of the right-hand side denote shorter RTs. Numbers on contour lines represent fitted inverse RT values.

**Figure 10.**
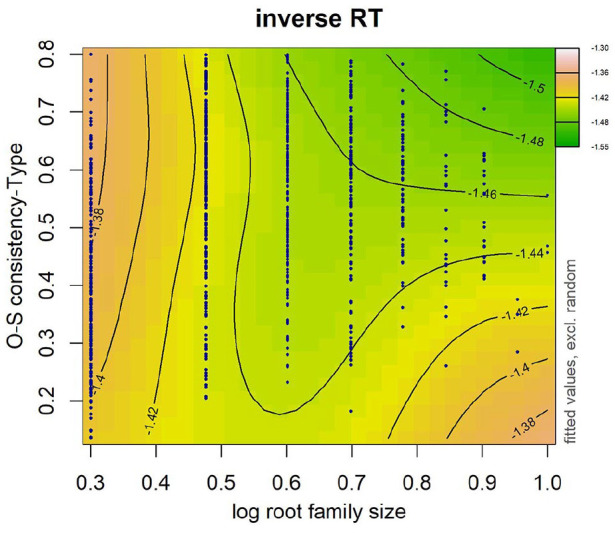
Word frequency was log transformed. Data are represented by dark blue points. Warmer colours (e.g., orange) on the left-hand side and bottom right-hand side denote longer RTs and cooler colours (e.g., green) on the top half of the right-hand side denote shorter RTs. Numbers on contour lines represent fitted inverse RT values.

Of interest is the first term after the tilde that accounts for the main effect of root family size and Orthographic-semantic and more importantly, the interaction between the two. In both models, the interaction between root family size and Orthographic-semantic was significant (see Table 14 for the model that included token-based Orthographic-semantic and Table 15 for type-based OSC). A facilitative effect of root family size was observed across a wide range of OSC estimates where data are available. On the contrary, a facilitative effect of OSC was only apparent in words with larger root families (see Figures 9 and 10). Response times were faster for words that were high in OSC than those lower in Orthographic-semantic, specifically for words that have larger morphological families. No effect of OSC was observed in words with very small root families. We reran an earlier model that had root family size, frequency, and the interaction between the two, except that this time the words that are highly consistent were excluded so that it could serve as a baseline model. Compared with this model, the model that had root family size, token-based OSC, and their interaction (∆AIC = 10.24; word frequency was controlled) was a better fit to the data. The model equivalent for type-based OSC (∆AIC = 4.93) was similarly a better fit to the data than the model without OSC and its interaction with root family size.

**Table 14.** Model output; OSC (Token) by root family size.

Parametric coefficients		Estimate	SE	*t*	*p*
	Intercept	−1.43	.02	−77.05	<.0001
	List two	.04	.01	4.28	<.0001
	List three	.03	.01	3.13	.002
	List four	−.02	.01	−1.56	.119
Approximate significance of smooth terms		*edf*	Ref. *df*	*F*	*P*
	Root family size, OSC (Token)	8.90	11.06	24.97	<.0001
	Frequency	5.98	7.16	293.24	<.0001
	Trial number, subjects	552.52	1,034.00	13.37	<.0001
*R*^2^ (adj.)	.39				
AIC	4,666.31				

OSC: orthographic–semantic consistency; AIC: Akaike information criterion.

*Note.* Word frequency and root family size were log transformed. The model syntax is inverse *RT* ~ *te*(root family size, OSC-Token) + *s*(frequency) + *list* + *s*(trial number, subjects, *bs* = “*fs*”, *m* = 1), data. OSC (Token) = orthographic-semantic consistency (Token).

**Table 15.** Model output; OSC (Type) by root family size.

Parametric coefficients		Estimate	SE	*t*	*p*
	Intercept	−1.43	.02	−77.21	<.0001
	List two	.04	.01	4.29	<.0001
	List three	.03	.01	3.07	.002
	List four	−.02	.01	−1.57	.117
Approximate significance of smooth terms		*edf*	Ref. *df*	*F*	*p*
	Root family size, OSC (Type)	7.97	9.86	27.68	<.0001
	Frequency	6.02	7.20	291.72	<.0001
	Trial number, subjects	555.28	1,034.00	13.41	<.0001
*R*^2^ (adj.)	.39				
AIC	4,663.40				

OSC: orthographic–semantic consistency; AIC: Akaike information criterion.

*Note.* Word frequency and root family size were log transformed. The model syntax is inverse *RT* ~ *te*(root family size, OSC-Type) + *s*(frequency) + *list* + *s*(trial number, subjects, *bs* = “*fs*”, *m* = 1), data. OSC (Type) = orthographic-semantic consistency (Type).

To conclude, it is not the case that Malay readers are impervious to orthographic relatives that do not share meaning. As illustrated in words with larger morphological families, response times are indeed slower for words that have low OSC (presumably because these words have many orthographic relatives that do not share meaning) than for words that have high OSC. In words with very small morphological families, however, OSC appeared to have little to no impact on Malay decision latencies. The significant interaction between family size and OSC complements the results from the model above that did not include this interaction. That is, OSC is a less crucial predictor of decision latencies than root family size as it seems to have an impact on Malay readers only for words with larger morphological families.

## Discussion

Two main objectives were accomplished in the present study. First, we expanded an existing Malay word database (i.e., MLP/MLP2) by adding a new measure (i.e., OSC) to the database that captures the consistency with which spelling patterns map onto a meaning. At the time of the present study, the MLP2 (a morphological version of the MLP database) listed the morphological family size for each word, a measure that estimates the number of words that share the same root. In a particular family, words that share a root are related in meaning. OSC, on the contrary, accounts for more fine-grained semantic information about a word by estimating how well the spelling of a word predicts its meaning. A pair of words that share spelling and meaning is assumed to be more closely related in a multidimensional semantic space than a word pair that shares an overlap in spelling but not meaning. Such a variable is useful for researchers to investigate the effects of spelling–meaning consistency in studies of Malay. We expanded the MLP2 by computing token-based and type-based OSC estimates for 2,287 monomorphemic words from the MLP/MLP2 database. This expansion is labelled as MLP3 with the intention that additional variables that capture other aspects of meaning in a word may be added to the database in future studies.

A second main goal of the study was to address a series of questions concerning the impact of root family size and OSC on Malay readers. To do so, we ran three sets of GAMMs. Whole-word frequency was accounted for in each model. First, we explored whether root family size and OSC were significant predictors of decision latencies in monomorphemic words, and whether these variables interacted with frequency. To address the first question, we ran a model each that included either root family size, token-based OSC, or type-based OSC as a predictor in the model. Root family size emerged as a significant predictor of Malay decision latencies in monomorphemic words. Root family size exerted a facilitative effect on decision latencies, that is, the larger the root families, the faster the responses. Our findings complement the effect of root family size reported in Marelli et al. (2015) for English monomorphemic words. As previously mentioned, this effect of root family size is well-established in prior studies of morphologically complex words in English (Ford et al., 2010; Sánchez-Gutiérrez et al., 2018), Danish (Balling & Baayen, 2008), Dutch (Bertram et al., 2000; de Jong et al., 2000), Estonian (Lõo et al., 2018), Finnish (Moscoso del Prado Martín et al., 2004), Hebrew (Moscoso del Prado Martin et al., 2005), and Malay (Maziyah Mohamed et al., 2023; Maziyah Mohamed & Jared, 2024). We found this effect of root family size to be particularly evident in lower frequency words. This interaction with frequency is analogous to that of Baayen et al. (2007) in which an effect of root family frequency (a token-count measure of root family size) was observed for lower frequency words in English.

With respect to OSC, both token-based and type-based OSC were significant predictors of Malay decision latencies. Like root family size, we found a facilitative effect of OSC. Across a wide range of OSC estimates, we observed faster responses the more consistently spelling patterns mapped onto a meaning. In addition, we found a significant interaction between OSC and frequency in that a facilitative effect of OSC is particularly apparent in lower frequency words. Somewhat surprising was the inhibitory effect of OSC for words that have very high OSC values. These words, however, represent a very small portion of our data. We suspect that OSC was not properly represented in this set of words because for most of these words the OSC estimate of a word was entirely reliant on one orthographic relative. Furthermore, we compared token-based and type-based OSC and evaluated whether one was a better predictor of Malay decision latencies than the other. It appears that both token-based and type-based OSC accounted for a similar amount of variance in Malay. This is perhaps not surprising as the two measures were very highly correlated here.

Then, we sought to establish whether there was an effect of OSC above and beyond family size on Malay decision latencies. We ran a second set of GAMMs that addressed whether an effect of OSC persists after accounting for root family size. Although initial analyses showed significant effects of OSC when the interaction of root family size and frequency was included in the model, those effects disappeared when we excluded the approximately 40 words that were highly consistent. That is, OSC did not account for additional variance when root family size was entered as a predictor in the same model. Root family size seems to be a more crucial predictor of Malay decision latencies than OSC. Recall that root family size critically differs from OSC in the types of orthographic neighbours that are included in their calculations. Orthographic neighbours that share spelling but not meaning are considered in the calculations of OSC but not root family size. One interpretation is that word neighbours that share spelling but not meaning (e.g., *ajar*—teach; *fajar*—dawn) do not have a large impact on Malay readers.

However, this initial interpretation did not entirely hold upon closer inspection. We next investigated whether there was a more fine-grained impact of OSC on Malay decision latencies. That is, we examined whether there was an interaction between root family size and OSC. Intriguingly, we found that an effect of OSC was only observed in words with a larger root family size. Responses were faster the more consistent the mappings were between spelling and meaning for words that have larger root families. No effect of OSC was observed in words with small root families. That is, word neighbours that share spelling but not meaning (e.g., *ajar*—teach; *fajar*—dawn) do have an impact on Malay readers, but primarily for words with large root families. In contrast, a facilitative effect of root family size was observed across a wide range of OSC values. In large root families, there are many orthographic neighbours that share an overlap in spelling. In those cases, orthographic information is perhaps not a very reliable cue for readers to distinguish among the words within a particular family. It is possible that in those cases readers pick up on some other word information (i.e., distributional properties, e.g., spelling-meaning consistency). To the best of our knowledge, there are no other studies that examine an interaction between OSC and root family size.

### Relation to previous studies

It is somewhat challenging to draw direct comparisons between the findings of the present study in Malay and those in the four studies in English that we have discussed (Hendrix & Sun, 2021; Marelli & Amenta, 2018; Marelli et al., 2015; Siegelman, Rueckl, et al., 2022) because each study used slightly different methodology in calculating OSC, and two did not include root family size in their models. However, despite the different languages, the results from this Malay study are fairly consistent with the English studies for comparable analyses. Marelli et al. found an effect of token-based OSC on English decision latencies after accounting for root family size. In that study, neighbours were defined as other words that begin with the same letter pattern. Marelli and Amenta (2018) then revised their definition of neighbours such that other words that contained the same letter pattern, regardless of position, were considered neighbours, and found this new OSC measure was an even better predictor of English lexical decision latencies. Here we used this expanded definition of neighbours, but we did not find a main effect of token-based OSC in Malay when root family size was accounted for, after excluding words that were highly consistent. Although our result appears to conflict with those of Marelli et al. (2015) and Marelli and Amenta (2018), this finding is similar to Siegelman, Rueckl, et al.’s (2022) observation of no effect of Marelli and Amenta’s token-based OSC after controlling for the problem that arises when the target word itself was considered a neighbour because we did not include the target word itself in the neighbourhood. Like Siegelman et al. and Hendrix and Sun (2021), we did find a main effect of type-based OSC on lexical decision latencies when root family size was not in the model, despite all three studies using different definitions of neighbours. However, models without root family size do not provide convincing evidence that OSC is distinct from the well-replicated effect of root family size. In our study, when root family size was in the model, we did not find a main effect of type-based OSC. A novel aspect of our analyses compared with the four other studies examining OSC effects that we have reviewed is that we investigated interactions of OSC with frequency and with root family size. OSC consistency effects were observed for Malay words with a larger root family size. In summary, it appears that both readers of Malay and readers of English pick up on the consistency with which letter patterns are mapped to meanings.

### Calculating OSC

Our review of four studies that have investigated OSC in English indicated that there has been a range of ways that researchers have calculated OSC. Studies have differed in how semantic vectors were generated, how orthographic neighbours were defined, and whether a type or token measure was used. OSC is a relatively new measure and ideas about how to calculate it are evolving. Next, we provide some thoughts on the calculation of OSC.

Creating semantic vectors is a complex process that requires considerable technical expertise. Fortunately, the fastText group provided pretrained word vectors (Wiki word vectors) developed using Bojanowski et al.’s (2017) prediction-based approach (fastText, skip-gram) for 294 languages, one of which was Malay. The existence of these vectors certainly made our task of calculating OSC for Malay much easier. Hendrix and Sun (2021) wrote favourably about the quality of semantic vectors generated using a fastText skip-gram model, particularly for lower frequency words, but as this approach is quite new, it is an open empirical question as to whether this method of creating vectors best captures the semantic representations of words. Bojanowski et al. (2017) provided some evidence that using subword *n*-grams improves semantic vector representations in the morphologically rich languages of German and Czech. They used *n*-grams of 3–6 letters in their work. In Grave et al.’s (2018) resource of semantic vectors for 157 languages created using fastText (CBOW), only *n*-grams of 5 letters were considered because this value produced faster training. Further work is needed to determine which choice of *n*-grams produces the best semantic vectors in Malay and other morphologically rich languages, and which method of generating semantic vectors (skip-gram or CBOW) better captures word meanings. For now, using Wiki word vector’s pretrained vectors seems to be a reasonable choice. We note that another option for creating semantic vectors for affixed stimuli that has appeared in a recent article (Bonandrini et al., 2023) is to combine semantic vector representations for affixes and stems; that is, to use explicit morphological information. However, the authors did not calculate OSC using these vectors.

An aspect of creating semantic vectors that we have not yet discussed concerns the size and source of the database from which the vectors were derived. Wikipedia is often used as a source. Grave et al. (2018) showed that for languages with a relatively small Wikipedia presence, adding language data from the common crawl web source improved the performance of the semantic vectors. Marelli and Amenta (2018) derived their semantic vectors from a dataset of 2.8 billion English tokens.

The choice of definition of neighbourhood likely has more of an impact on OSC values. As we noted, Siegelman, Rueckl, et al. (2022) made a strong argument as to why the word itself should not be included in its neighbourhood. Marelli and Amenta (2018) provided evidence that the definition of neighbourhood that produced the strongest OSC effect on English reading times was one in which neighbours were words that embed the word of interest. That is, they were neighbours that had the target word plus one or more letters. We based our neighbourhood definition on these two ideas. Other definitions of neighbourhood that Marelli and Amenta explored were based on Levenshtein distance (e.g., 10, 20, or 30 shortest LD, or LD of 1, 2, or 3) and these produced weaker OSC effects. Levenshtein distance includes three types of word neighbours: those that are formed by adding one or more letters to the target, those formed by changing one or more letters in the target, and those formed by deleting one or more letters from the target. The number of letters changed indicates the Levenshtein distance. It may be the case that not all of these types of neighbours are equally influential. Hino et al. (2023) demonstrated a significant effect of OSC on English lexical decisions when neighbours were defined as words that had the target plus one more letter, but not when neighbours were words that substituted one target word letter. Both Siegelman, Rueckl, et al. (2022) and Hendrix and Sun (2021) used a Levenshtein distance measure to define neighbours (LD1 and 5 shortest LD, respectively) and found significant effects of OSC on reading times. It is possible that addition of neighbours drove these effects, but that is an open empirical question. It also needs to be determined how many letters with different neighbours can be and still contribute to improving the calculation of OSC. Here and in Marelli and Amenta’s (2018) work, neighbour words included the target word and any number of extra letters. Restricting the definition of neighbours to include only a single additional letter, as LD1 does, would miss any morphological relatives that had prefixes and suffixes that were longer than a single letter.

Another aspect of the OSC calculation is whether a type or token measure should be used. We were unable to shed light on this issue here because these were highly correlated in our dataset. Further research needs to explore this parameter.

### Experimental task

OSC provides a quantitative measure of the strength of mappings between spelling and sound. This measure predicted response times in the lexical decision task which does not have an explicit semantic requirement. The lexical decision task was chosen here partly to fit with existing studies of OSC and partly for ease of data collection. Future work could explore whether OSC is an even stronger predictor of performance in a reading task that has a more obvious semantic requirement. Siegelman, Rueckl, et al. (2022) provided evidence that their OSC measure predicted gaze durations on data from the GECO book reading corpus (Cop et al., 2017), which was produced by participants whose eyes were tracked while reading an English novel for comprehension. Surprisingly, the partial *R*^2^ for OSC was actually smaller in their eye-tracking data than in their lexical decision data. There is currently no comparable source of eye-tracking data for Malay, but there is a source, the Multilingual Eye-Movements Corpus (Siegelman, Schroeder, et al., 2022), that has eye movement data from readers of other agglutinating languages including Estonian, Finnish, Korean, and Turkish as well as nine other languages. Participants at each site read 12 passages, 5 of which had the same content translated. These data may be useful for researchers of these other agglutinating languages who wish to explore OSC.

### Theoretical implications

The findings of the present study fit with the distributed approach to morphological processing (Baayen et al., 2019; Marelli & Baroni, 2015; Plaut & Gonnerman, 2000; Rueckl & Raveh, 1999) that assumes that readers learn statistical co-occurrences between spelling and meaning. In a distributed account, the representation of a word’s form and meaning are shaped by the distributional properties of the language, including the distributional properties of roots (e.g., DLM; Baayen et al., 2019).

For example, in a connectionist model (e.g., Rueckl & Raveh, 1999) weights on connections between orthographic units and hidden units, and between hidden units and semantic units are initially set to small random values (for simplicity we will ignore the phonological pathway to meaning). On each learning trial, the model is presented with a word, and activation flows from orthographic units through to semantic units, producing a pattern of activation across each set of units. A learning algorithm then adjusts the weights on each set of connections slightly so that a more accurate pattern of activation is produced on the semantic units the next time the same word is presented to the orthographic units. Because the same sets of units and connections are used in processing every word, the performance on each word is affected by exposure to the other words in the training set. Words that are similarly spelled and that have similar meanings (e.g., root family members such as *bright, brighter, brightest, brightly, brightness*) will have similar effects on the weights; therefore, exposure to one word improves performance on the others. This process would give rise to root family size effects. Words that are orthographically similar but semantically dissimilar drive the weights in competing directions; that is, training on *whisker* would have a negative impact on the weights for *whiskey* and vice versa. The net effect of the entire ensemble of learning experiences is poorer performance on words in inconsistent neighbourhoods compared with words in consistent neighbourhoods, or an effect of OSC. The factor that has the biggest impact on the model’s performance on a particular word is exposure to the word itself. For this reason, effects of root family size and OSC would be expected to be smaller for high-frequency words than for low-frequency words, as was observed here.

Rueckl (2010) noted that morphological structure creates islands of regularities in the otherwise arbitrary mapping from orthography to semantics. When Rueckl and Raveh (1999) examined the hidden layer representations in the model that they trained on a language that contained morphological regularities, they observed a clustering of morphological relatives in the hidden layer of the model even though no explicit morphological information was given to the model. This finding indicates that hidden unit representations become organised to capture similarities among both the orthographic input patterns and similarities among the semantic units to which these inputs must be mapped. Probing the representations on a hidden layer of units in a connectionist model that is trained on either small or large morphological families might help us understand why only words with large morphological families were affected by OSC in the present study. After training, Rueckl and Raveh observed that roots and suffixes were represented by distinct hidden layer subpatterns, indicating that multimorphemic words had a componential structure. The hidden layer subpatterns for roots likely developed with exposure to more and more different members of the same root family. It might be the case that these subpatterns have to develop to a certain extent before they are influenced by neighbours that share some spelling but have a different meaning. It might also be the case that the extent to which an inconsistent neighbour influences the strength of weights for a root word depends on whether the inconsistent neighbour has a pseudoaffix (e.g., *whisk-er*) or not (e.g., *bask-et*). The representation of the latter may be less likely to overlap with the subpattern for a real root (e.g., *bask*).

The data provided here, and in our two other publications on Malay (Maziyah Mohamed et al., 2023; Maziyah Mohamed & Jared, 2024), provide information on how distributional properties of morphemes influence word reading times that will be needed to develop and test a distributional model of Malay word recognition. Creating such a model was beyond the scope of the current study, but is in the future plans for this programme of research. Our studies suggest that research even in English needs to pay greater attention to interactions among distributional variables, particularly whether they interact with whole-word frequency, and whether effects of OSC depend on root family size. More clarity on these issues will also be useful in testing computational models that are currently available. Specifically, if the interaction between OSC and morphological family size is also observed in English, such a finding would be an interesting focus of simulations using existing computational models and could perhaps be a “benchmark” effect that can distinguish between them (see Amenta & Crepaldi, 2012 for a discussion of the importance of “benchmark” effects for the development of models of morphological processing).

An important implication of the assumption that readers pick up on the distributional properties of their language is that because distributional properties may differ in their range of values across languages, the degree to which these properties impact word reading times in each language may differ (see Laudanna & Burani, 1995). To ensure that theoretical models reflect language processing in general, we need to have research on readers’ sensitivity to various distributional properties from a variety of languages and not rely exclusively on studies of English. Conducting studies in other languages (including other agglutinative languages) will help provide some clarity as to whether agglutinating languages typically differ from other languages in the relative strength of root family size and OSC effects. The semantic vectors compiled by the fastText team for words in many languages could be a very helpful resource for such studies.

### Future directions for the MLP

On our wish list to be added to the MLP3 are other distributional properties that can capture spelling–meaning consistency in affixes, and a list of semantic variables that quantify various aspects of word meaning. Currently available in the MLP2/MLP3 are prefix and suffix consistency measures that estimate the number of words in which a letter string functions as an affix divided by the total number of words that contain a particular letter string. This measure of affix consistency, however, does not take into account the number of meanings that each affix carries. For example, some prefixes in Malay are more consistent in their function than others. The prefix *ter–* is typically used as a superlative (e.g., *terbesar/largest*) or to describe actions that were accidental (e.g., *tertumpah/spill*) and the prefix *pe*– often marks for occupations (e.g., *pelakon/actor*), whereas other prefixes such as *peN-* carry a wider variety of meanings (Denistia & Baayen, 2019). A measure of OSC for affixes would provide a more sensitive measure to estimate the reliability of an affix letter pattern as a cue to word meaning.

Semantic variables such as imageability (Bennett et al., 2011; Binder et al., 2005; Yap et al., 2012), number of features (Hargreaves & Pexman, 2014; McRae et al., 2005; Pexman et al., 2002, 2003, 2008; Yap et al., 2011, 2012), body–object interaction (Bennett et al., 2011; Yap et al., 2012), number of senses (Rodd et al., 2002; Yap et al., 2012), number of associates (Pexman et al., 2007), and semantic neighbourhood size or density (Buchanan et al., 2001; Pexman et al., 2008; Yap et al., 2011, 2012), and contextual diversity (Adelman et al., 2006) or contextual distinctiveness (Hoffman et al., 2013; McDonald & Shillcock, 2001) have contributed much to the research of reading in English. Such a list of norms could be collected for Malay words in the MLP database.

While the number of words in the MLP is sizable, future steps may include expanding the size of the MLP corpus. The calculations of distributional properties often depend on the size of the corpus, making it a challenge to conduct reliable cross-language comparisons using large corpora that differ greatly in their sizes (e.g., the ELP is seven times larger in size than the MLP). An initial step in increasing the number of words in the MLP can be done by extracting a list of Malay words in existing sources. Open Lexicon (http://www.lexique.org/shiny/openlexicon/) has word frequencies for many languages, including Malay such as the WorldLex-Malaysian resource that contains over 300,000 words.

### Conclusion

The purpose of the present study was twofold: to expand an existing large Malay database by calculating OSC estimates for 2,287 words and adding new lexical decision latencies for 1,280 words (a total of 3,777 words in the MLP3 have lexical decision latencies in addition to 1,520 words that already have lexical decision latencies in the original MLP), and to extend the research on the impact of OSC on reading times to Malay readers. MLP3 can be accessed using the following link: https://osf.io/dhyzb/?view_only=e22e226ac62c4fd2b54de5d27467404e. Concerning the effect of OSC on Malay decision latencies, a key takeaway is that Malay readers are impacted by the consistency of the mappings between spelling and meaning but only for words that have larger root families. Distributional models of Malay word processing will need to account for these effects of OSC, root family size, and particularly the interaction between the two. In conclusion, we hope that researchers who are interested in Malay word recognition find these OSC estimates useful in future Malay reading experiments. We invite researchers to contribute measures of other characteristics of Malay words to continue building this resource.

## Supplemental Material

sj-docx-1-qjp-10.1177_17470218241234668 – Supplemental material for Malay Lexicon Project 3: The impact of orthographic–semantic consistency on lexical decision latencies Supplemental material, sj-docx-1-qjp-10.1177_17470218241234668 for Malay Lexicon Project 3: The impact of orthographic–semantic consistency on lexical decision latencies by Mirrah Maziyah Mohamed and Debra Jared in Quarterly Journal of Experimental Psychology
