# Supplementary material for: Malay Lexicon Project 3: The impact of orthographic–semantic consistency on lexical decision latencies
Source: Q J Exp Psychol (Hove). 2024 Mar 21;78(1):22–47. doi: 10.1177/17470218241234668 (PMC11684139; doi:10.1177/17470218241234668)
Supplement: sj-docx-1-qjp-10.1177_17470218241234668 – Supplemental material for Malay Lexicon Project 3: The impact of orthographic–semantic consistency on lexical decision latencies [file sj-docx-1-qjp-10.1177_17470218241234668.docx]

Supplementary Materials for:

**Malay Lexicon Project 3: The Impact of Orthographic-Semantic Consistency on Lexical Decision Latencies**

Mirrah Maziyah Mohamed and Debra Jared

Department of Psychology, University of Western Ontario

**Author Note**

Mirrah Maziyah Mohamed
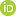
 https://orcid.org/0000-0002-3164-3805

Debra Jared
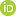
 https://orcid.org/0000-0002-5818-4119

This work was supported by a Natural Sciences and Engineering Research Council (NSERC) of Canada Discovery Grant to D.J. We would like to express our sincere gratitude to Andrew Mitchell for calculating OSC estimates for a large set of Malay words, Melvin J. Yap for sharing his list of Malay pseudowords, and Harald Baayen for taking the time to teach us the statistical techniques used in the present study.

Correspondence concerning this article should be directed to M. Maziyah Mohamed, mmoha87@uwo.ca or Debra Jared, djjared@uwo.ca. Western Interdisciplinary Research Building, University of Western Ontario, 1151 Richmond St., London, ON, Canada, N6A 3K7.

**Table S1**

*Model Concurvity (Root family size)*

|  | Parametric | Word frequency | Root Family Size | Trial number, Subjects |
| --- | --- | --- | --- | --- |
| Worst | 1 | .09 | .81 | 1.00 |
| Observed | 1 | .06 | .15 | .05 |
| Estimate | 1 | .06 | .45 | .00 |

*Note.* Word frequency and root family size were log transformed. The model syntax here is inverse RT ~ s(log word frequency) + s(log root family size) + list + s(trial number, subject, bs=”fs”, m=1), data.

**Figure S1**

*Diagnostic Plots (Root family size)*

**
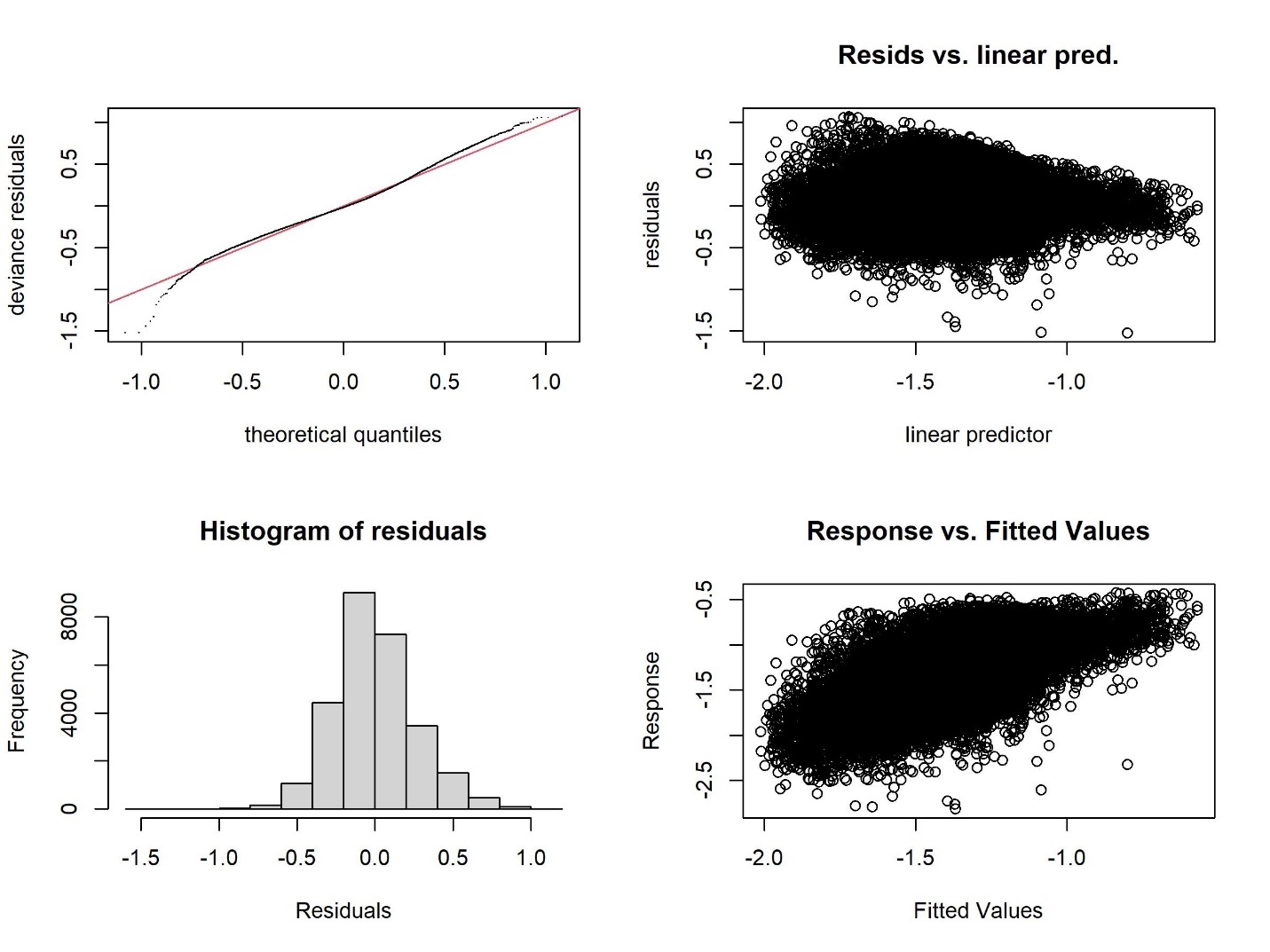
**

*Note.* Top row: Q-Q plot indicating model residuals follow a normal distribution indicated by the two overlapping straight lines (left), and the plot of residual values evenly distributed around zero (right). Bottom row: Symmetrical bell curved histogram of residuals (left), and the plot of response against fitted values clustered around a 1 to -1 line (right). The model syntax here is inverse RT ~ s(log word frequency) + s(log root family size) + list + s(trial number, subject, bs=”fs”, m=1), data.

**Table S2**

*Model Concurvity (Root family size*Frequency)*

|  | Parametric | Word frequency, Root Family Size | Trial number, Subjects |
| --- | --- | --- | --- |
| Worst | 1 | .95 | 1.00 |
| Observed | 1 | .06 | .05 |
| Estimate | 1 | .09 | .00 |

*Note.* Word frequency and root family size were log transformed. The model syntax here is inverse RT ~ te(log word frequency, log root family size) + list + s(trial number, subject, bs=”fs”, m=1), data.

**Figure S2**

*Diagnostic Plots (Root family size*Frequency)*


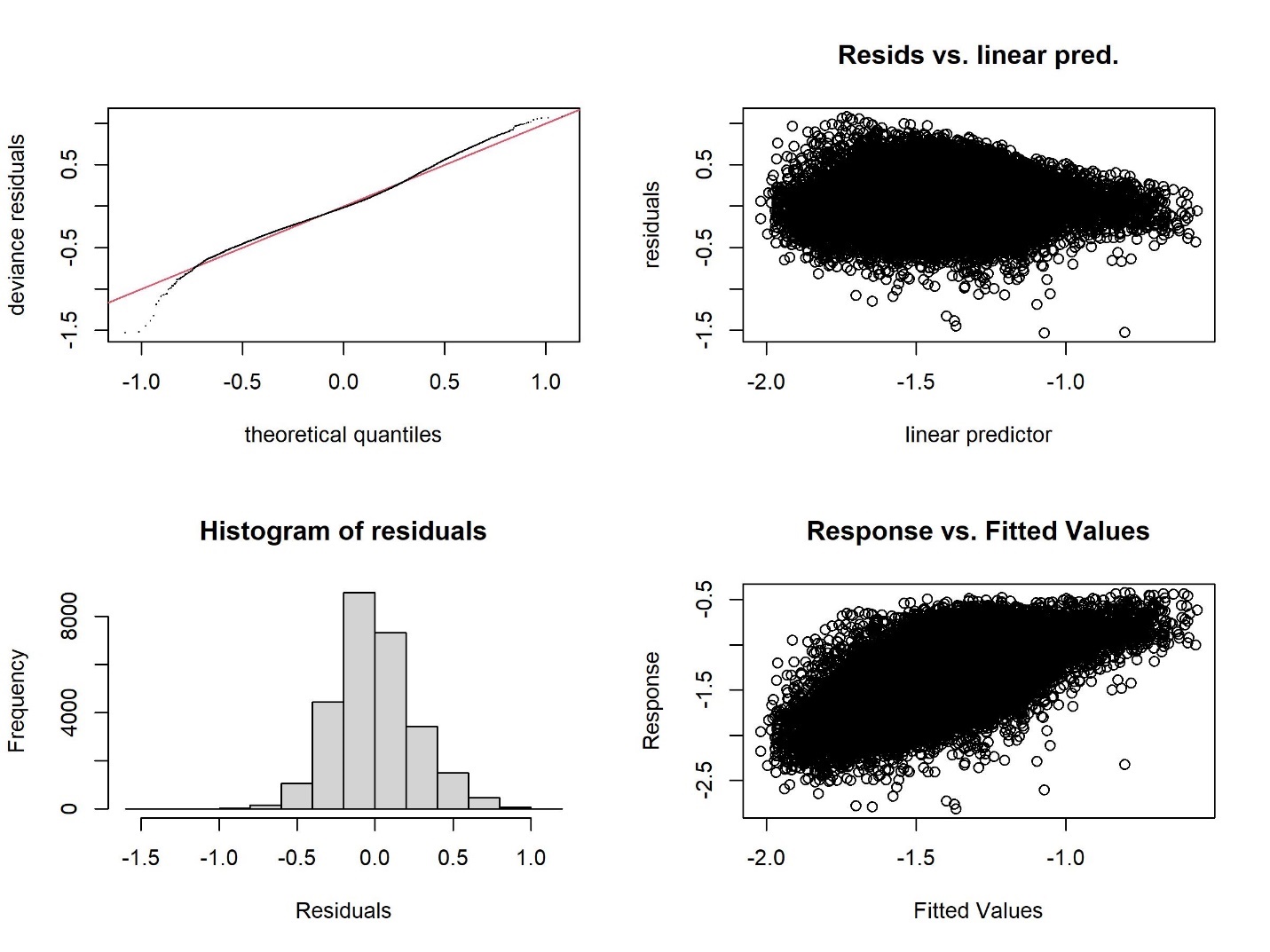


*Note.* Top row: Q-Q plot indicating model residuals follow a normal distribution indicated by the two overlapping straight lines (left), and the plot of residual values evenly distributed around zero (right). Bottom row: Symmetrical bell curved histogram of residuals (left), and the plot of response against fitted values clustered around a 1 to -1 line (right). The model syntax here is inverse RT ~ te(log word frequency, log root family size) + list + s(trial number, subject, bs=”fs”, m=1), data.

**Table S3**

*Model Concurvity (OSC-Token)*

|  | Parametric | Word frequency | OSC-Token | Trial number, Subjects |
| --- | --- | --- | --- | --- |
| Worst | 1 | .17 | .16 | 1.00 |
| Observed | 1 | .15 | .07 | .05 |
| Estimate | 1 | .14 | .11 | .00 |

*Note.* Word frequency was log transformed. OSC=Orthographic-Semantic Consistency. The model syntax here is inverse RT ~ s(log word frequency) + s(OSC-Token) + list + s(trial number, subject, bs=”fs”, m=1), data.

**Figure S3**

*Diagnostic Plots (OSC-Token)*

**
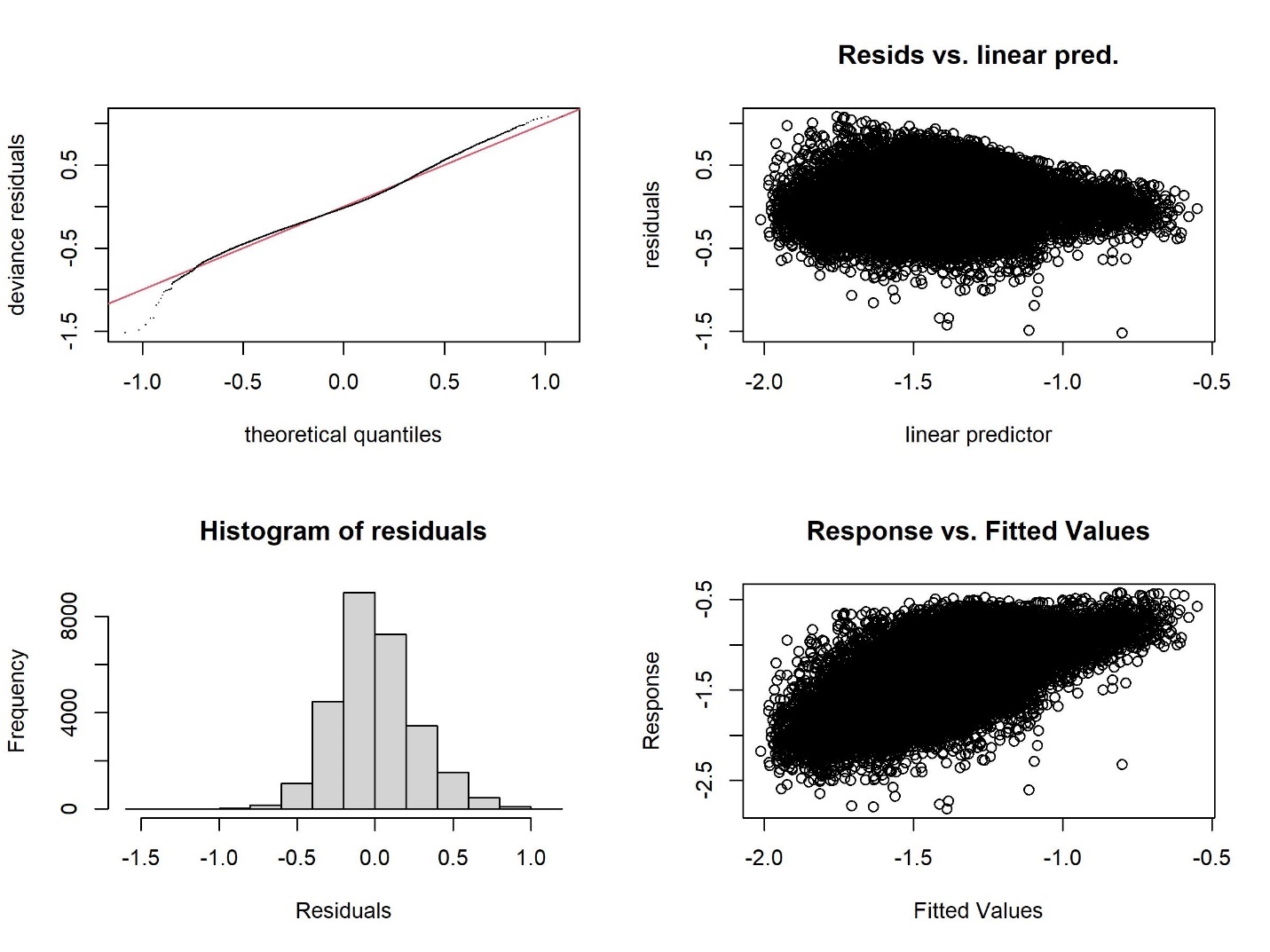
**

*Note.* Top row: Q-Q plot indicating model residuals follow a normal distribution indicated by the two overlapping straight lines (left), and the plot of residual values evenly distributed around zero (right). Bottom row: Symmetrical bell curved histogram of residuals (left), and the plot of response against fitted values clustered around a 1 to -1 line (right). The model syntax here is inverse RT ~ s(log word frequency) + s(OSC-Token) + list + s(trial number, subject, bs=”fs”, m=1), data.

**Table S4**

*Model Concurvity (OSC-Type)*

|  | Parametric | Word frequency | OSC-Type | Trial number, Subjects |
| --- | --- | --- | --- | --- |
| Worst | 1 | .17 | .16 | 1.00 |
| Observed | 1 | .15 | .08 | .05 |
| Estimate | 1 | .14 | .11 | .00 |

*Note.* Word frequency was log transformed. OSC=Orthographic-Semantic Consistency. The model syntax here is inverse RT ~ s(log word frequency) + s(OSC-Type) + list + s(trial number, subject, bs=”fs”, m=1), data.

**Figure S4**

*Diagnostic Plots (OSC-Type)*


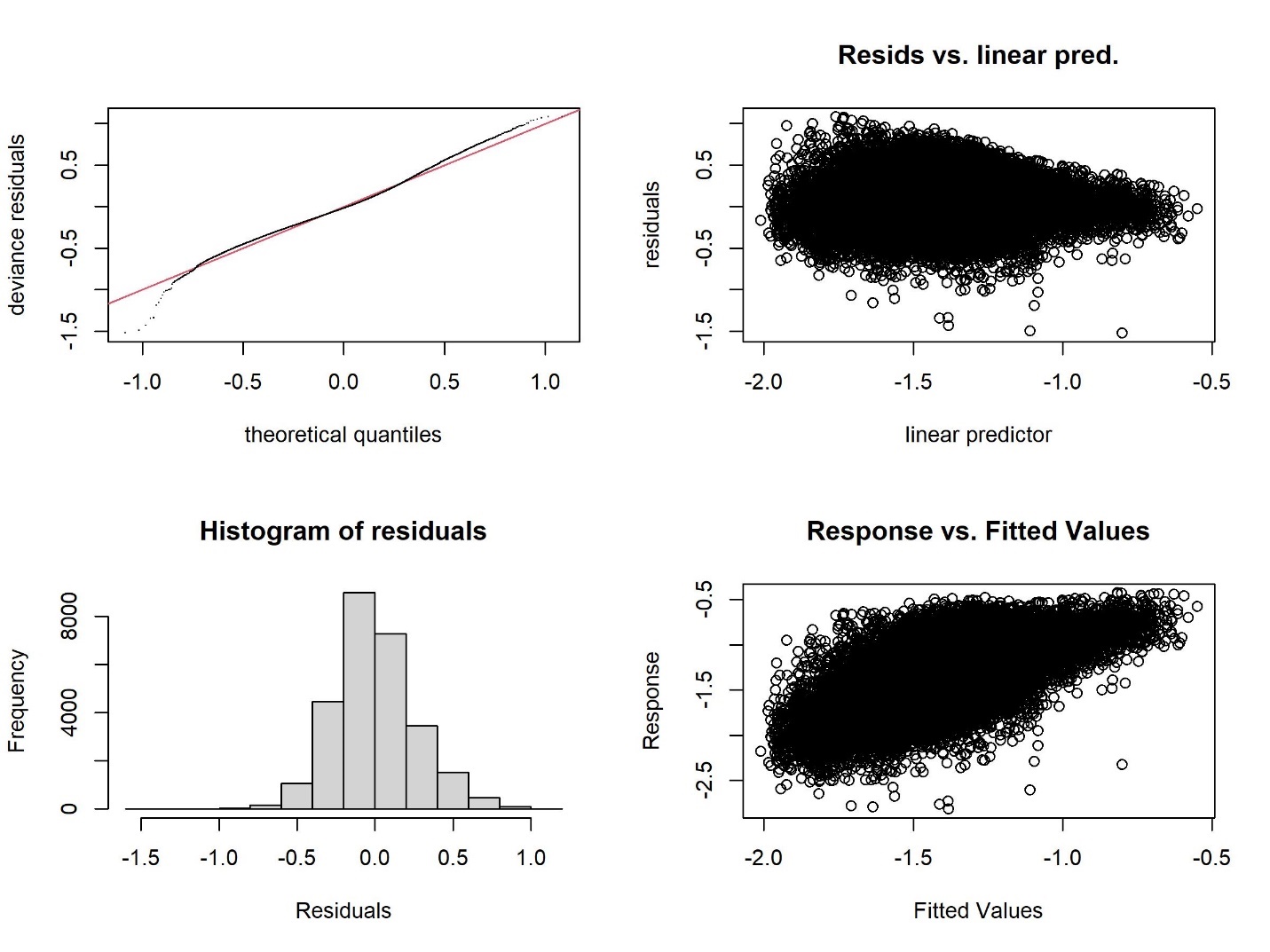


*Note.* Top row: Q-Q plot indicating model residuals follow a normal distribution indicated by the two overlapping straight lines (left), and the plot of residual values evenly distributed around zero (right). Bottom row: Symmetrical bell curved histogram of residuals (left), and the plot of response against fitted values clustered around a 1 to -1 line (right). The model syntax here is inverse RT ~ s(log word frequency) + s(OSC-Type) + list + s(trial number, subject, bs=”fs”, m=1), data.

**Table S5**

*Model Concurvity (OSC-Token*Frequency)*

|  | Parametric | Word frequency, OSC-Token | Trial number, Subjects |
| --- | --- | --- | --- |
| Worst | 1 | .18 | 1.00 |
| Observed | 1 | .04 | .05 |
| Estimate | 1 | .05 | .00 |

*Note.* Word frequency was log transformed. OSC=Orthographic-Semantic Consistency. The model syntax here is inverse RT ~ te(log word frequency, OSC-Token) + list + s(trial number, subject, bs=”fs”, m=1), data.

**Figure S5**

*Diagnostic Plots (OSC-Token*Frequency)*


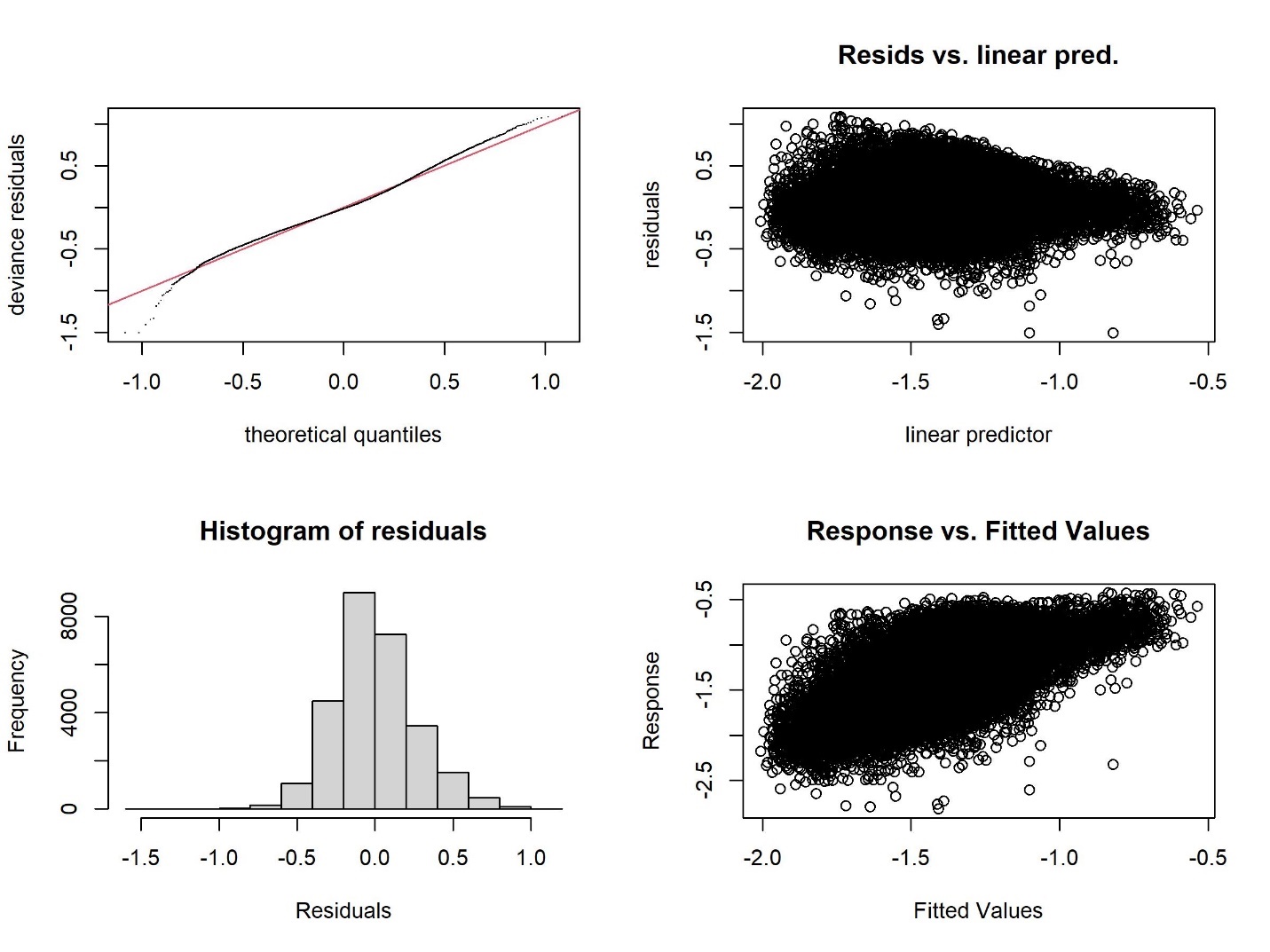


*Note.* Top row: Q-Q plot indicating model residuals follow a normal distribution indicated by the two overlapping straight lines (left), and the plot of residual values evenly distributed around zero (right). Bottom row: Symmetrical bell curved histogram of residuals (left), and the plot of response against fitted values clustered around a 1 to -1 line (right). The model syntax here is inverse RT ~ te(log word frequency, OSC-Token) + list + s(trial number, subject, bs=”fs”, m=1), data.

**Table S6**

*Model Concurvity (OSC-Type*Frequency)*

|  | Parametric | Word frequency, OSC-Type | Trial number, Subjects |
| --- | --- | --- | --- |
| Worst | 1 | .19 | 1.00 |
| Observed | 1 | .04 | .05 |
| Estimate | 1 | .05 | .00 |

*Note.* Word frequency was log transformed. OSC=Orthographic-Semantic Consistency. The model syntax here is inverse RT ~ te(log word frequency, OSC-Type) + list + s(trial number, subject, bs=”fs”, m=1), data.

**Figure S6**

*Diagnostic Plots (OSC-Type*Frequency)*


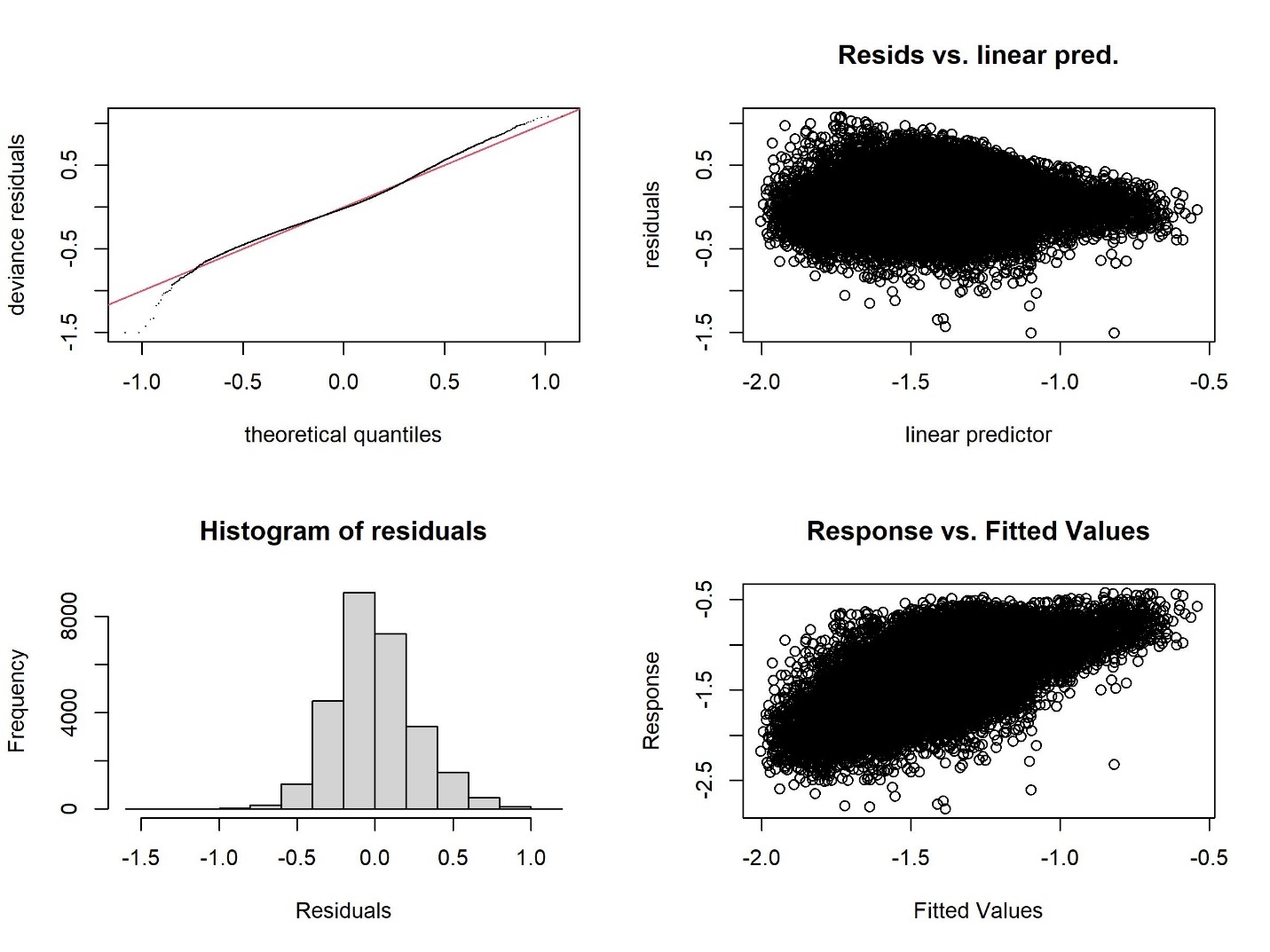


*Note.* Top row: Q-Q plot indicating model residuals follow a normal distribution indicated by the two overlapping straight lines (left), and the plot of residual values evenly distributed around zero (right). Bottom row: Symmetrical bell curved histogram of residuals (left), and the plot of response against fitted values clustered around a 1 to -1 line (right). The model syntax here is inverse RT ~ te(log word frequency, OSC-Type) + list + s(trial number, subject, bs=”fs”, m=1), data.

**Table S7**

*Model Concurvity (Root Family Size and OSC-Token)*

|  | Parametric | Word frequency, Root Family Size | OSC-Token | Trial number, Subjects |
| --- | --- | --- | --- | --- |
| Worst | 1 | .96 | .30 | 1.00 |
| Observed | 1 | .14 | .11 | .05 |
| Estimate | 1 | .13 | .26 | .00 |

*Note.* Word frequency and root family size were log transformed. OSC=Orthographic-Semantic Consistency. The model syntax here is inverse RT ~ te(log word frequency, log root family size) + s(OSC-Token) + list + s(trial number, subject, bs=”fs”, m=1), data.

**Figure S7**

*Diagnostic Plots (Root Family Size*Frequency + OSC-Token)*


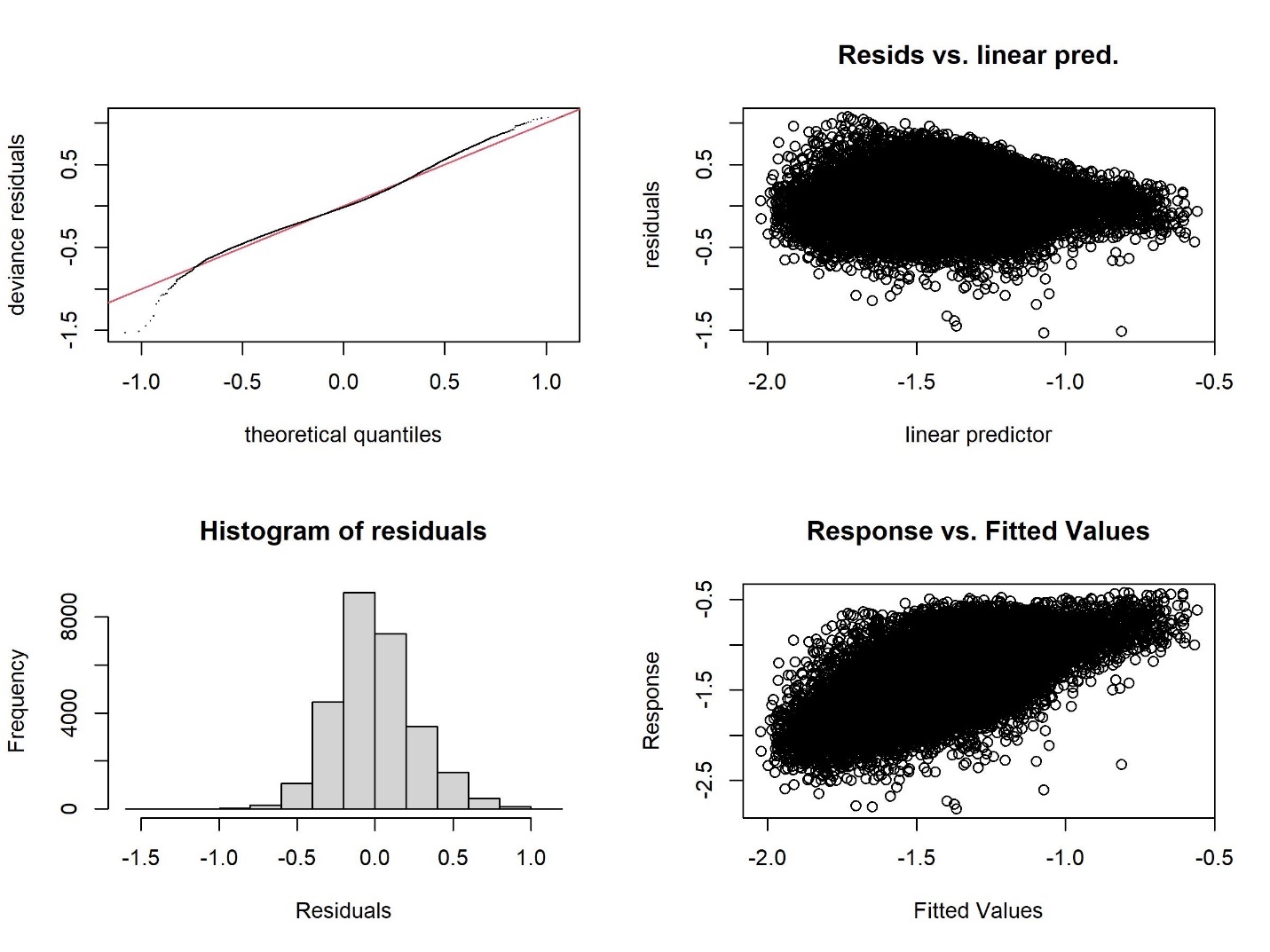


*Note.* Top row: Q-Q plot indicating model residuals follow a normal distribution indicated by the two overlapping straight lines (left), and the plot of residual values evenly distributed around zero (right). Bottom row: Symmetrical bell curved histogram of residuals (left), and the plot of response against fitted values clustered around a 1 to -1 line (right). The model syntax here is inverse RT ~ te(log word frequency, log root family size) + s(OSC-Token) + list + s(trial number, subject, bs=”fs”, m=1), data.

**Table S8**

*Model Concurvity (Root Family Size and OSC-Type)*

|  | Parametric | Word frequency, Root Family Size | OSC-Type | Trial number, Subjects |
| --- | --- | --- | --- | --- |
| Worst | 1 | .96 | .32 | 1.00 |
| Observed | 1 | .14 | .10 | .05 |
| Estimate | 1 | .13 | .27 | .00 |

*Note.* Word frequency and root family size were log transformed. OSC=Orthographic-Semantic Consistency. The model syntax here is inverse RT ~ te(log word frequency, log root family size) + s(OSC-Type) + list + s(trial number, subject, bs=”fs”, m=1), data.

**Figure S8**

*Diagnostic Plots (Root Family Size*Frequency + OSC-Type)*


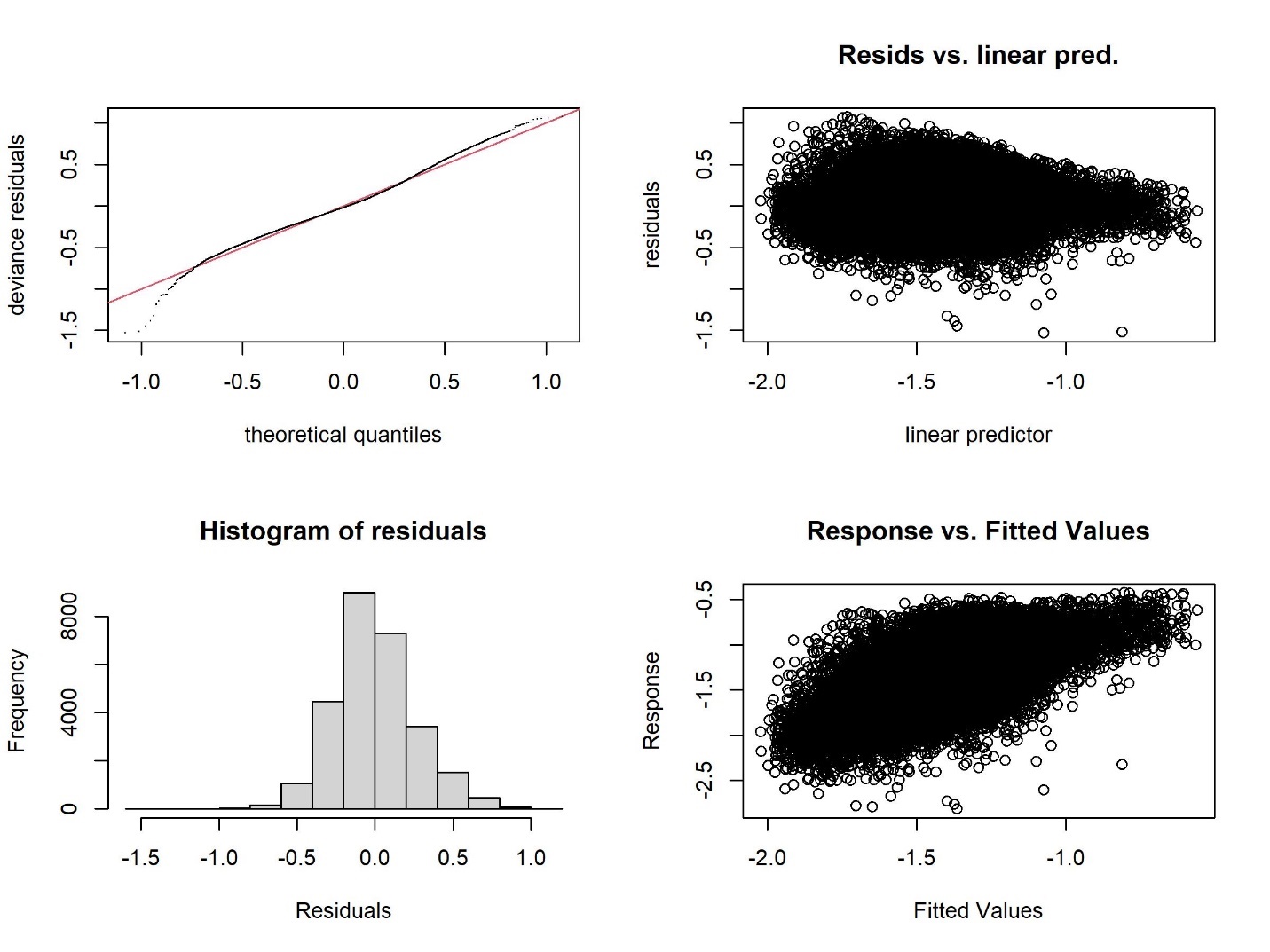


*Note.* Top row: Q-Q plot indicating model residuals follow a normal distribution indicated by the two overlapping straight lines (left), and the plot of residual values evenly distributed around zero (right). Bottom row: Symmetrical bell curved histogram of residuals (left), and the plot of response against fitted values clustered around a 1 to -1 line (right). The model syntax here is inverse RT ~ te(log word frequency, log root family size) + s(OSC-Type) + list + s(trial number, subject, bs=”fs”, m=1), data.

**Table S9**

*Model Concurvity (Root Family Size and OSC-Token)*

|  | Parametric | Word frequency, Root Family Size | OSC-Token | Trial number, Subjects |
| --- | --- | --- | --- | --- |
| Worst | 1 | .97 | .30 | 1.00 |
| Observed | 1 | .13 | .18 | .05 |
| Estimate | 1 | .13 | .26 | .00 |

*Note.* This dataset excludes approximately 40 words that have higher OSC estimates (OSC≥.8). Word frequency and root family size were log transformed. OSC=Orthographic-Semantic Consistency. The model syntax here is inverse RT ~ te(log word frequency, log root family size) + s(OSC-Token) + list + s(trial number, subject, bs=”fs”, m=1), data.

**Figure S9**

*Diagnostic Plots (Root Family Size*Frequency + OSC-Token)*


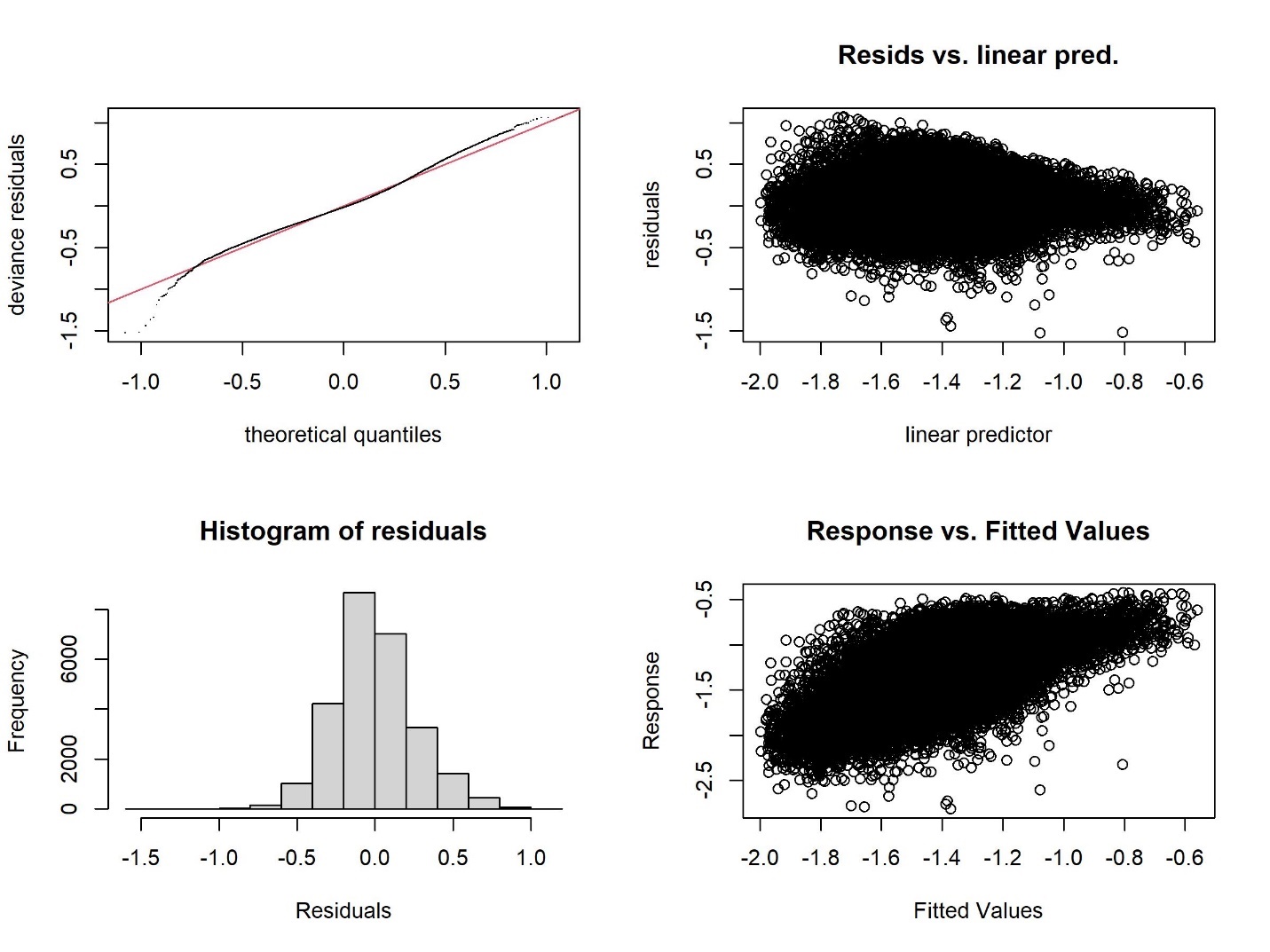


*Note.* This dataset excludes approximately 40 words that have higher OSC estimates (OSC≥.8). Top row: Q-Q plot indicating model residuals follow a normal distribution indicated by the two overlapping straight lines (left), and the plot of residual values evenly distributed around zero (right). Bottom row: Symmetrical bell curved histogram of residuals (left), and the plot of response against fitted values clustered around a 1 to -1 line (right). The model syntax here is inverse RT ~ te(log word frequency, log root family size) + s(OSC-Token) + list + s(trial number, subject, bs=”fs”, m=1), data.

**Table S10**

*Model Concurvity (Root Family Size and OSC-Type)*

|  | Parametric | Word frequency, Root Family Size | OSC-Type | Trial number, Subjects |
| --- | --- | --- | --- | --- |
| Worst | 1 | .96 | .32 | 1.00 |
| Observed | 1 | .13 | .13 | .05 |
| Estimate | 1 | .13 | .28 | .00 |

*Note.* This dataset excludes approximately 40 words that have higher OSC estimates (OSC≥.8). Word frequency and root family size were log transformed. OSC=Orthographic-Semantic Consistency. The model syntax here is inverse RT ~ te(log word frequency, log root family size) + s(OSC-Type) + list + s(trial number, subject, bs=”fs”, m=1), data.

**Figure S10**

*Diagnostic Plots (Root Family Size*Frequency + OSC-Type)*


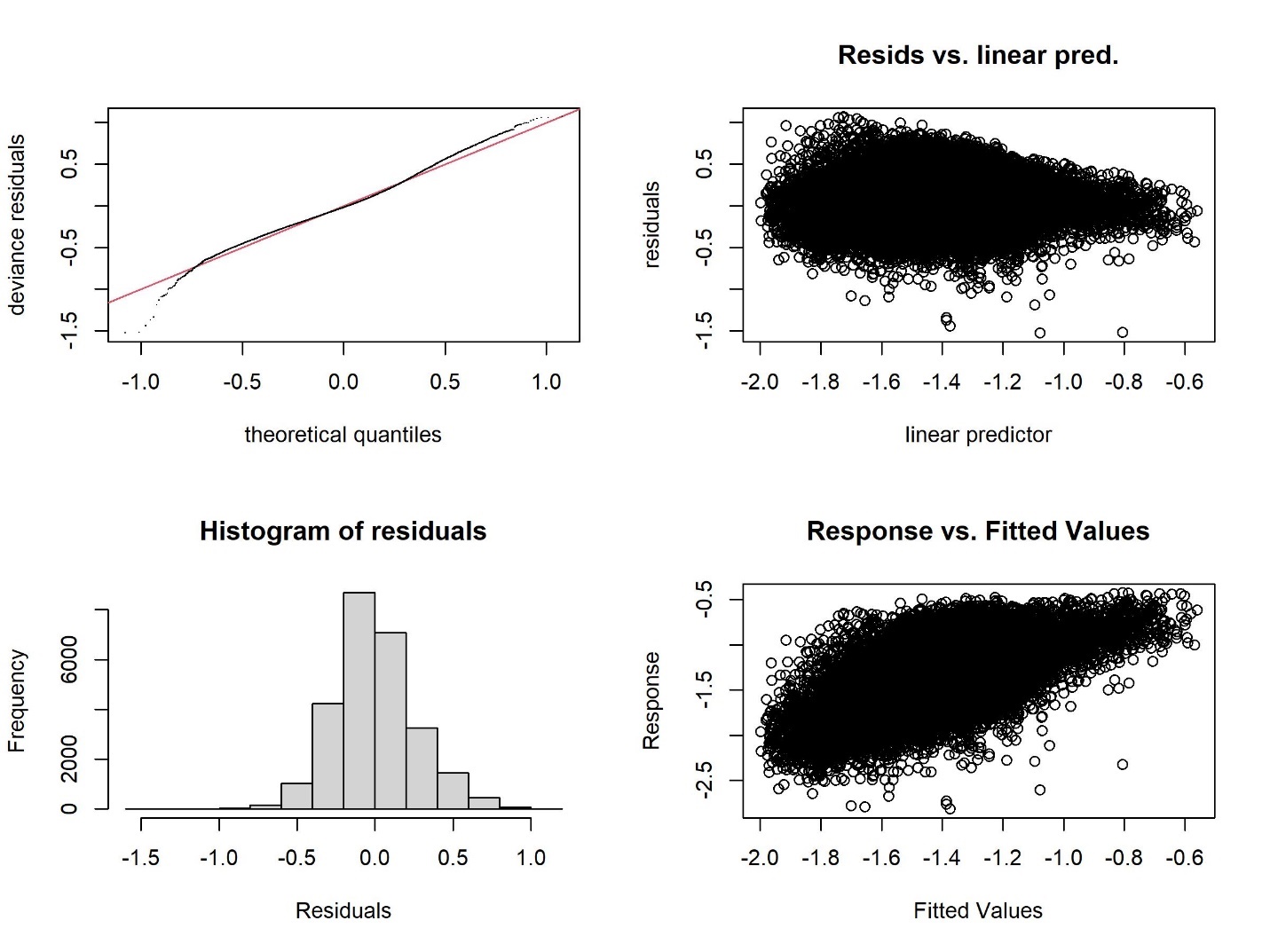


*Note.* This dataset excludes approximately 40 words that have higher OSC estimates (OSC≥.8). Top row: Q-Q plot indicating model residuals follow a normal distribution indicated by the two overlapping straight lines (left), and the plot of residual values evenly distributed around zero (right). Bottom row: Symmetrical bell curved histogram of residuals (left), and the plot of response against fitted values clustered around a 1 to -1 line (right). The model syntax here is inverse RT ~ te(log word frequency, log root family size) + s(OSC-Type) + list + s(trial number, subject, bs=”fs”, m=1), data.

**Table S11**

*Model Concurvity (Root Family Size*OSC-Token)*

|  | Parametric | Word frequency | OSC-Token, Root Family Size | Trial number, Subjects |
| --- | --- | --- | --- | --- |
| Worst | 1 | .23 | .99 | 1.00 |
| Observed | 1 | .20 | .14 | .05 |
| Estimate | 1 | .19 | .13 | .00 |

*Note.* This dataset excludes approximately 40 words that have higher OSC estimates (OSC≥.8). Word frequency and root family size were log transformed. OSC=Orthographic-Semantic Consistency. The model syntax here is inverse RT ~ s(log word frequency) + te(log root family size, OSC-Token) + list + s(trial number, subject, bs=”fs”, m=1), data.

**Figure S11**

*Diagnostic Plots (Root Family Size*OSC-Token)*

*
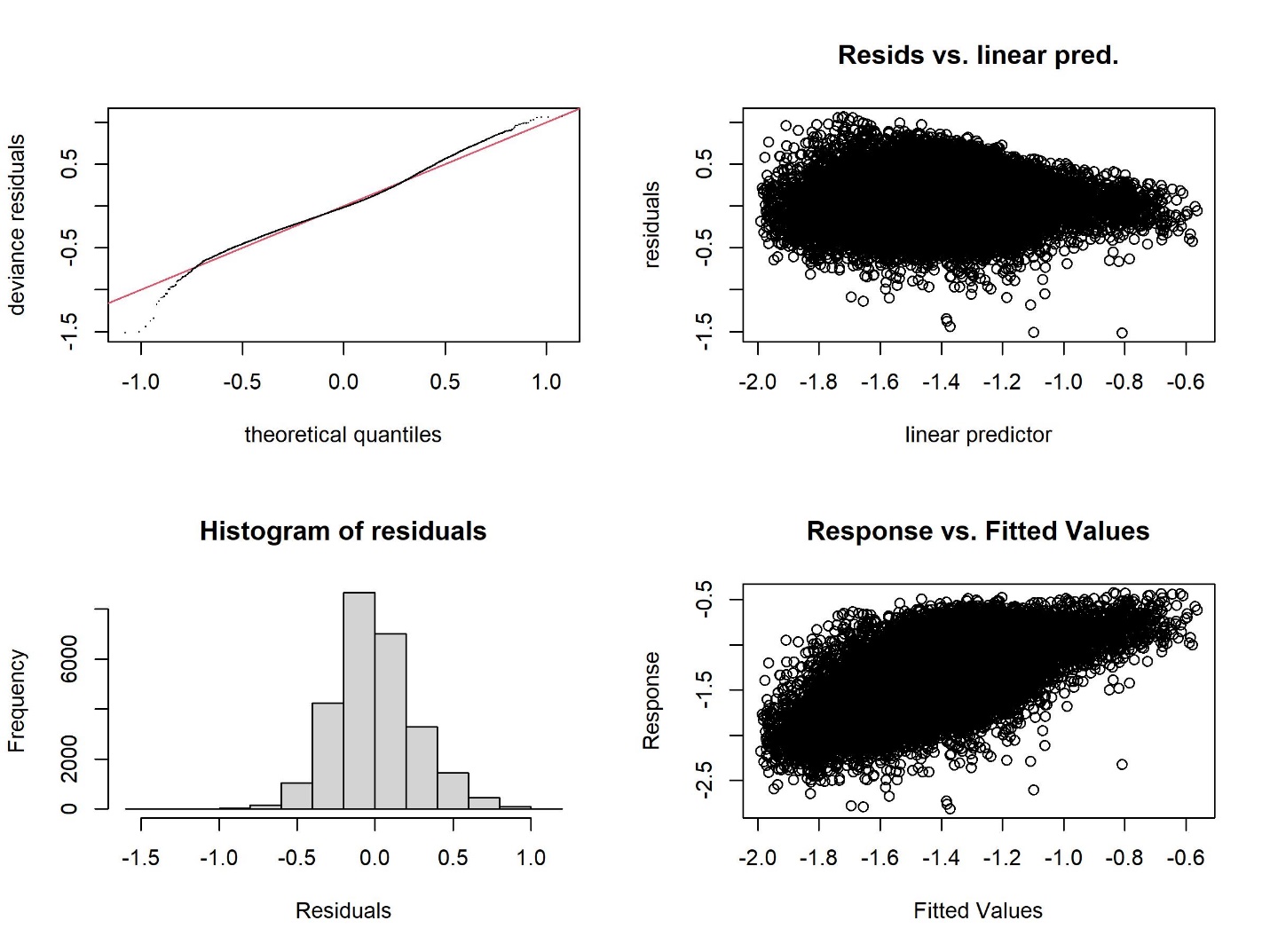
*

*Note.* This dataset excludes approximately 40 words that have higher OSC estimates (OSC≥.8). Top row: Q-Q plot indicating model residuals follow a normal distribution indicated by the two overlapping straight lines (left), and the plot of residual values evenly distributed around zero (right). Bottom row: Symmetrical bell curved histogram of residuals (left), and the plot of response against fitted values clustered around a 1 to -1 line (right). The model syntax here is inverse RT ~ s(log word frequency) + te(log root family size, OSC-Token) + list + s(trial number, subject, bs=”fs”, m=1), data.

**Table S12**

*Model Concurvity (Root Family Size*OSC-Type)*

|  | Parametric | Word frequency | OSC-Type, Root Family Size | Trial number, Subjects |
| --- | --- | --- | --- | --- |
| Worst | 1 | .23 | .99 | 1.00 |
| Observed | 1 | .21 | .13 | .05 |
| Estimate | 1 | .20 | .13 | .00 |

*Note.* This dataset excludes approximately 40 words that have higher OSC estimates (OSC≥.8). Word frequency and root family size were log transformed. OSC=Orthographic-Semantic Consistency. The model syntax here is inverse RT ~ s(log word frequency) + te(log root family size, OSC-Type) + list + s(trial number, subject, bs=”fs”, m=1), data.

**Figure S12**

*Diagnostic Plots (Root Family Size*OSC-Type)*


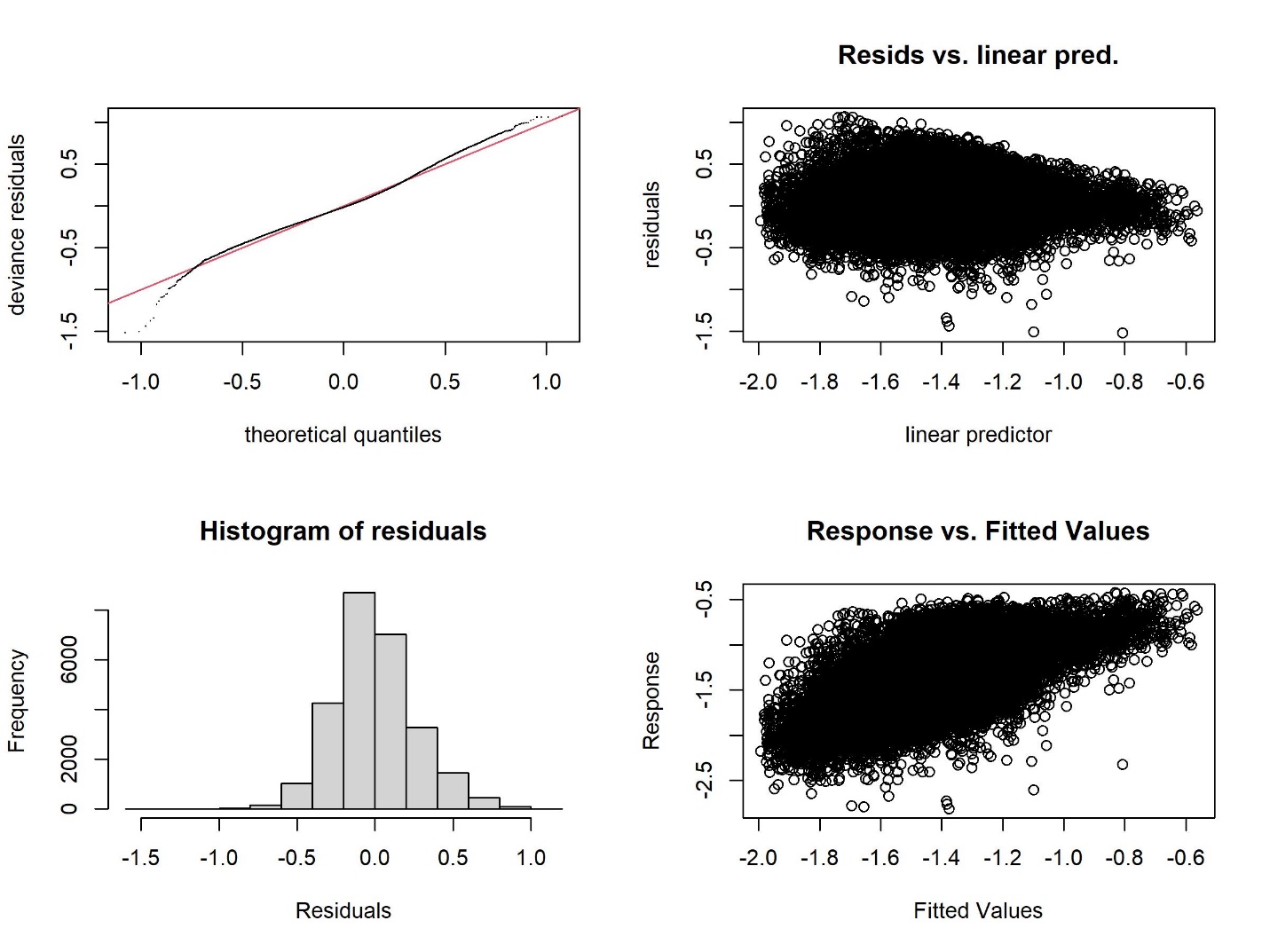


*Note.* This dataset excludes approximately 40 words that have higher OSC estimates (OSC≥.8). Top row: Q-Q plot indicating model residuals follow a normal distribution indicated by the two overlapping straight lines (left), and the plot of residual values evenly distributed around zero (right). Bottom row: Symmetrical bell curved histogram of residuals (left), and the plot of response against fitted values clustered around a 1 to -1 line (right). The model syntax here is inverse RT ~ s(log word frequency) + te(log root family size, OSC-Type) + list + s(trial number, subject, bs=”fs”, m=1), data.
